# Impact of *C-* and *N-*terminal protection on the stability, metal chelation and antimicrobial properties of calcitermin

**DOI:** 10.1038/s41598-023-45437-0

**Published:** 2023-10-25

**Authors:** Maria D’Accolti, Denise Bellotti, Emilia Dzień, Carlotta Leonetti, Silvia Leveraro, Valentina Albanese, Erika Marzola, Remo Guerrini, Elisabetta Caselli, Magdalena Rowińska-Żyrek, Maurizio Remelli

**Affiliations:** 1 Department of Chemical, Pharmaceutical and Agricultural Sciences, University of Ferrara, L. Borsari 46, 44121 Ferrara, Italy; 2 Faculty of Chemistry, University of Wrocław, F. Joliot-Curie 14, 50-383 Wrocław, Poland; 3 Department of Environmental and Prevention Sciences, University of Ferrara, L. Borsari 46, 44121 Ferrara, Italy

**Keywords:** Coordination chemistry, Peptides, Metals, Antimicrobials

## Abstract

The main limitation to the use of antimicrobial peptides (AMPs) as regular drugs, against antibiotic and antifungal resistance, mainly relates to their rapid degradation by proteolytic enzymes. The introduction of suitable structural changes in the peptide chain can make the peptide less susceptible to the action of proteases, thus overcoming this problem. To improve the plasma stability of calcitermin, a metal-chelating AMP present in the human respiratory tract and investigated in the present study, *C-* and/or *N-* terminal modifications have been introduced in the native sequence. Evaluation of peptide stability has been performed to determine the half-life times in human plasma of both native calcitermin and its derivatives. However, the protection of the peptide termini can also affect its metal coordination behaviour. Thus, the characterization of Zn^2+^ and Cu^2+^ complexes has been performed by means of several techniques, including potentiometry, high-resolution mass spectrometry, UV–Vis, circular dichroism and EPR. On the basis of the obtained results, it was possible to compare the biological activity of the studied systems, taking into account both the metal-binding ability and the peptide stability to search for a link among them. A significant result of this study is that the *N-*terminal protection increases the calcitermin half-life over seven times and the formation of metal complexes confers resistance towards degradation almost doubling its half-life.

## Introduction

Antimicrobial resistance (AMR) is one of the most important health challenges of the twenty-first century. Among several novel approaches aimed at overcoming the global drug-resistance crisis, the use of antimicrobial peptides (AMPs) represents a promising strategy for the design of new drugs^1^. They exhibit a broad spectrum of activity, being effective against a wide variety of pathogens, like Gram-positive^2^ and Gram-negative bacteria^3^, fungi^4^, viruses^5^ and even cancer cells^5^ and are present in all living organisms (invertebrates, vertebrates, plants, prokaryotes)^6^. However, these extraordinary candidate molecules present some drawbacks, the major one being the fact that they are metabolically unstable. The AMPs activity level is subject to modulation or degradation by both human and pathogenic proteolytic enzymes. In fact, many endo- and exo-peptidases act to transform high molecular weight peptides into shorter oligopeptides, making them inactive. This problem translates into short half-lives (usually less than 30 min) and scarce bioavailability^7^. Therefore, in order to improve the therapeutic potential of these molecules, a rational design of new AMP analogues is required with the aim of optimizing their chemical properties and the engineering of delivery systems^8^.

Calcitermin is a 15-mer peptide (VAIALKAAHYHTHKE, **WT**) corresponding to the *C-*terminal domain of calgranulin C, a pro-inflammatory protein of the S100 family. It contains an effective metal-binding domain with three alternated histidine residues in position 9, 11 and 13, and the free terminal amino and carboxyl groups^9^. The complex-formation equilibria of this natural peptide with Zn^2+^ and Cu^2+^ ions have been recently studied by our research group to obtain information on stoichiometry and structure of complex species formed throughout a wide pH range^9^. Moreover, calcitermin proved to adopt a helical conformation in membranes, and Zn^2+^ and Cu^2+^ ions are able to enhance its antimicrobial activity against *C. albicans* and common bacteria like *S. aureus* and *E. faecalis*^9–11^.

In order to improve the microbicide activity of calcitermin, different strategies can be employed: *(i)* extending its life-time, i.e. its resistance to proteases; *(ii)* enhancing its sequestering ability towards the metal micronutrients (necessary for the survival and virulence of pathogens), thus hampering the microbe growth; *(iii)* changing the peptide charge and structure before and/or after the metal interaction^12,13^. It is worth to note that three calcitermin His-to-Ala mutants − H9A, H11A and H13A, where one histidine residue is replaced with alanine—exhibit promising antimicrobial activity according to the estimated minimal inhibitory concentrations (MIC) in vitro^9^. These calcitermin mutants indeed represent an outstanding example of how different metal coordination modes (obtained by means of His-to-Ala substitution) result in significant changes of their antimicrobial properties. This type of information can be obtained through a detailed investigation on thermodynamics and coordination chemistry of the metal-peptide interaction, which can lay the foundations for a deeper insight into the way of action of metal chelating AMPs, in order to design new effective antibiotic therapies.

Considering the above results, this work is aimed at finding calcitermin derivatives where the peptide structure and, consequently, its physico-chemical properties are modified in order to achieve, first of all, a longer half-life and a lower proteolytic susceptibility, without losing its antimicrobial properties. Several strategies to improve the stability of peptides have been explored in the last forty years^14^. To increase the resistance towards exo-peptidases one common strategy is based on the chemical modification of one or both the peptide ends (*e.g.* by* N*-acylation, *C*-amidation, cyclization, binding to polyethyleneglycol)^8^. The degradation by endo-peptidases can be instead prevented by replacing one or more residues with non-proteinogenic amino acids which are normally not recognized by enzymes, like D-amino acids^15^, *N*-methyl-α-amino acids^16^, β- and γ- amino acids^17–19^, α-alkylated amino acids^20^, *N*-substituted glycines^21^.

Given the importance of the peptide termini in the degradation processes carried out by exopeptidases—enzymes that catalyze the cleavage of the terminal peptide bonds—we protected the amino- and carboxyl-termini of calcitermin by acetylation and amidation respectively^22^, obtaining the following derivatives: Ac-VAIALKAAHYHTHKE (**L1**), Ac-VAIALKAAHYHTHKE-NH_2_ (**L2**) and VAIALKAAHYHTHKE-NH_2_ (**L3**). The terminal protection should confer higher proteolytic stability and preserve the principal metal binding site of calcitermin, corresponding to the histidine motif -HxHxH-. Moreover, the study of the protected peptides will elucidate the role of the terminal groups in calcitermin antimicrobial and metal-chelating properties. An in-depth investigation on the complex-formation equilibria of these derivatives with Zn^2+^ and Cu^2+^ ions, together with their stability and activity, will provide further information on their way of action.

## Results and discussion

Under the experimental conditions here employed, only variously protonated mononuclear complexes, with metal/ligand stoichiometry of 1:1, have been detected by potentiometry and mass spectrometry. No precipitation has been observed over the explored pH range (2.5–10.5). The overall complex-formation constants (log*β*) and corresponding acid dissociation constants (p*K*_a_) are reported in Tables 1, 2 and Tables S1, S2, and the calculated species distribution diagrams for each system are shown in Figs. S1–S8. Spectroscopic results, including Vis absorption spectra, CD spectra and EPR spectra, recorded at different pH values, are shown in Figs. S9–S20.

**Table 1:** Equilibrium constants and proposed coordination modes for Cu^2+^ complexes at *T* = 298 K and *I* = 0.1 M (KCl).

Species	Ac-VAIALKAAHYHTHKE (**L1**)			Ac-VAIALKAAHYHTHKE-NH_2_ (**L2**)		
	log*β*	p*K*_a_	Coordination	log*β*	p*K*_a_	Coordination
**[CuH**_**2**_**L]**^**4+**^	18.21(3)	4.40	N_Im_, COO^–^	23.06(4)	5.49	2N_Im_
**[CuHL]**^**3+**^	13.81(2)	5.38	2N_Im_, COO^–^	17.57(5)	6.30	3N_Im_
**[CuL]**^**2+**^	8.43(2)	6.59	3N_Im_	11.27(7)	6.70	3N_Im_, N^–^
**[CuH**_**-1**_**L]**^**+**^	1.84(3)	6.88	3N_Im_, N^–^	4.57(6)	9.44	2N_Im_, 2N^–^
**[CuH**_**-2**_**L]**	− 5.04(3)	–	2N_Im_, 2N^–^	− 4.87(9)	9.91	2N_Im_, 2N^–^
**[CuH**_**-3**_**L]**^**–**^	–	–	–	− 14.78(7)	–	N_Im_, 3N^–^

Species	VAIALKAAHYHTHKE-NH_2_ (**L3**)					
	log*β*	p*K*_a_	Coordination			
**[CuH**_**5**_**L]**^**5+**^	51.38(2)	5.16	2N_Im_			
**[CuH**_**4**_**L]**^**4+**^	46.21(2)	6.21	3N_Im_			
**[CuH**_**3**_**L]**^**3+**^	40.00(3)	6.90	2N_Im_, NH_2_			
**[CuH**_**2**_**L]**^**2+**^	33.11(2)	7.75	2N_Im_, NH_2_, N^–^			
**[CuHL]**^**+**^	25.36(3)	9.53	2N_Im_, 2N^–^			
**[CuL]**	15.83(5)	10.01	2N_Im_, 2N^–^			
**[CuH**_**-1**_**L]**^**–**^	5.82(4)	–	N_Im_, 3N^–^			
**[CuH**_**-3**_**L]**^**3–**^	− 15.03(9)	–	N_Im_, 3N^–^			

Values in parentheses are standard deviations on the last significant figure.

**Table 2:** Equilibrium constants and proposed coordination modes for Zn^2+^ complexes at *T* = 298 K and *I* = 0.1 M (KCl).

Species	Ac-VAIALKAAHYHTHKE (**L1**)			Ac-VAIALKAAHYHTHKE-NH_2_ (**L2**)		
	log*β*	p*K*_a_	Coordination	log*β*	p*K*_a_	Coordination
**[ZnH**_**2**_**L]**^**4+**^	–	–	–	20.29(6)	6.14	2N_Im_
**[ZnHL]**^**3+**^	11.00(5)	5.80	2N_Im_	14.15(4)	7.63	3N_Im_
**[ZnL]**^**2+**^	5.21(2)	7.33	3N_Im_	6.52(5)	8.93	3N_Im_, OH^–^
**[ZnH**_**-1**_**L]**^**+**^	− 2.12(3)	–	3N_Im_, OH^–^	− 2.41(7)	9.44	3N_Im_, 2OH^–^
**[ZnH**_**-2**_**L]**				− 11.85(3)	–	3N_Im_, 2OH^–^

Species	VAIALKAAHYHTHKE-NH_2_ (**L3**)					
	log*β*	p*K*_a_	Coordination			
**[ZnH**_**5**_**L]**^**5+**^	48.7(1)	5.6	2N_Im_			
**[ZnH**_**4**_**L]**^**4+**^	43.07(3)	7.32	3N_Im_			
**[ZnH**_**3**_**L]**^**3+**^	35.75(6)	7.69	3N_Im_, OH^–^			
**[ZnH**_**2**_**L]**^**2+**^	28.06(4)	8.65	3N_Im_, OH^–^			
**[ZnHL]**^**+**^	19.40(5)	9.47	3N_Im_, 2OH^–^			
**[ZnL]**	9.94(4)	–	3N_Im_, 2OH^–^			
**[ZnH**_**-2**_**L]**^**2–**^	− 10.70(7)	–	3N_Im_, 2OH^–^			

Values in parentheses are standard deviations on the last significant figure.

### Formation of copper complexes with L1, L2 and L3

ESI–MS results are reported in Table S4 and Figs. S21–S23: the only detected *m/z* signals correspond to equimolar Cu^2+^ complexes with different protonation states. Potassium and/or sodium adducts are also formed. The formation of copper complexes in solution begins around pH 3–3.5. The first detected species, however, display different coordination modes depending on the system.

**L1** is the only ligand with the free carboxyl terminus, and its first formed species, [CuH_2_L]^4+^, is likely characterized by a coordination mode (N_Im_, COO^–^). In this case, besides the histidine residue, one or both the carboxyl groups of *C-*terminal glutamic acid can participate in the complexation, forming a macrocycle. The wavelength of maximum absorption measured at pH 4, at least partly imputable to the species [CuH_2_L]^4+^ (760 nm, Fig. S9), is in fair agreement with the expected value for a coordination (N_Im_, COO^–^)^23^, taking into account that at this pH most of the copper is still present as a hexa-aquo complex. The complex [CuH_2_L]^4+^ is rapidly substituted in solution by the species [CuHL]^3+^, where a second histidine interacts with the metal center (p*K*_a_ = 4.40) giving a (2N_Im_, COO^–^) complex (experimental *λ*_max_ = 655 nm, Fig. S9; predicted* λ*_max_ = 663 nm^23^). In the case of amidated peptides (**L2** and **L3**), the stoichiometry of the complex species formed at the most acidic pH values ([CuH_2_L]^4+^ and [CuH_5_L]^5+^, respectively) suggests that two histidines are already deprotonated. In other words, the protection of the *C-*terminus likely favors the initial formation of a 2N complex where Cu^2+^ interacts with two imidazole groups of the histidine side chains. Indeed, the 2N species are predominant in solution in the pH range 4–5.5. EPR spectra registered at pH 5.5 confirm the presence of 2N complexes (**L1**: A_//_ = 174; g_//_ = 2.28; g_⊥_ = 2.02; **L2**: A_//_ = 157; g_//_ = 2.31; g_⊥_ = 2.02; **L3**: A_//_ = 163; g_//_ = 2.28; g_⊥_ = 2.02; Table S3)^24^. Increasing the pH value, the Cu^2+^ ion can displace the acidic proton from a third histidine, and it coordinates three imidazoles forming a (3N_Im_) species, which is prevalent in solution at around pH 6 in all the systems. The obtained wavelengths of maximum absorption at pH 6.0 range from *λ*_max_ = 626 nm to *λ*_max_ = 635 nm and agree with the proposed coordination mode (expected *λ*_max_ = 627 nm)^23^.

In the alkaline pH range, we can distinguish two situations depending on the considered system. In the case of acetylated peptides, **L1** and **L2**, the next deprotonation step involves an amide group of the peptide backbone, while in **L3**, the free terminal amino group can deprotonate and interact with the metal ion. The EPR results confirm the formation of 4N species, since the spectra above pH 7 show the same superhyperfine structure and the following EPR parameters: A_//_ = 182–198; g_//_ = 2.20–2.21; g_⊥_ = 2.02–2.05 (Table S3), that agree with the expected literature values^24^.

**Figure 1:**
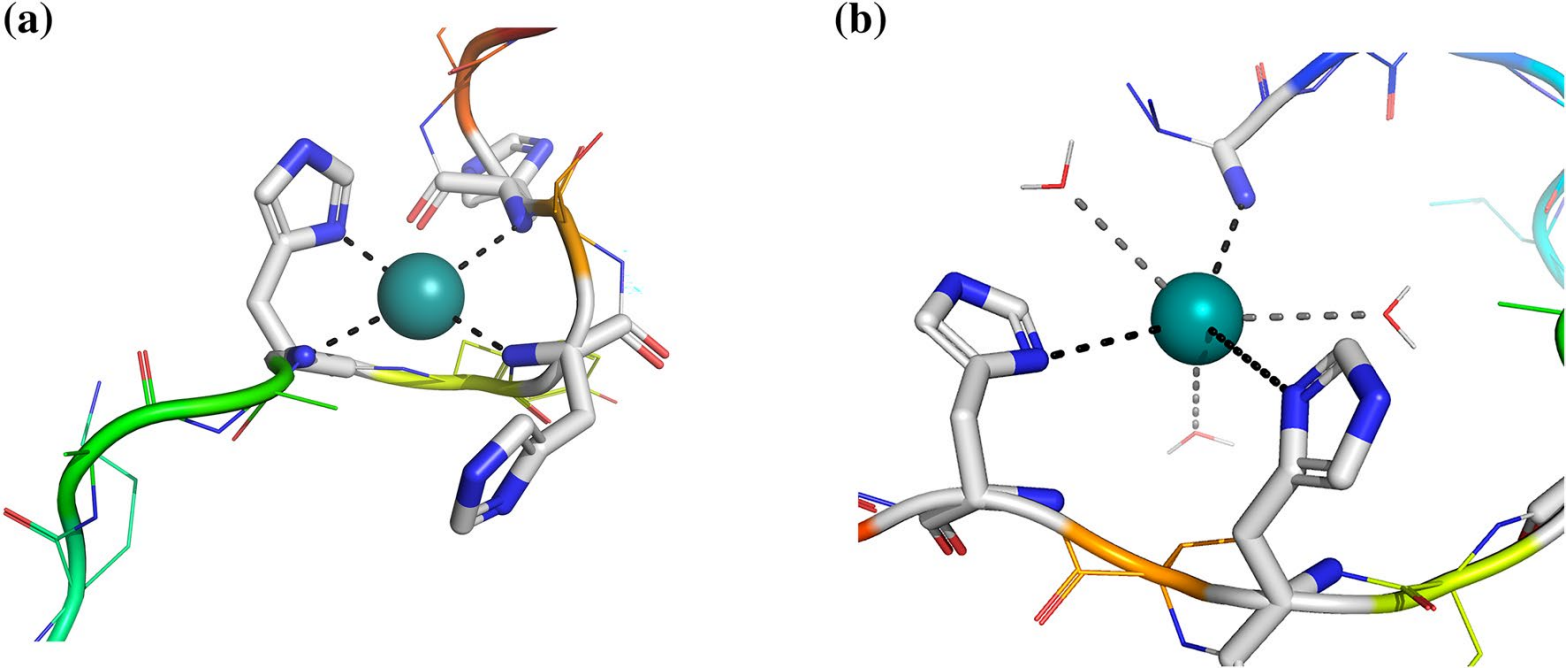
(**a**) Coordination hypothesis (N_Im_, 3N^–^) for [CuH_-3_L]^–^ species of peptide **L2**. (**b**) Coordination hypothesis (2N_Im_, NH_2_) for the [CuH_3_L]^3+^ species of peptide **L3**. Molecular structure generated with PyMol^27^.

Examining in details the systems **L1** and **L2**, starting from pH 5.5, we can observe the formation of [CuH_-1_L]^+^ (for **L1**) and [CuL]^2+^ (for **L2**) (Figs. S3, S4) with p*K*_a_ = 6.59 and 6.30, respectively. The experimental values of *λ*_max_ for solutions where these complexes reach their maximum of formation is 557 nm (Figs. S9, S12), exactly the value expected for a coordination (3N_Im_, N^–^)^23^. Circular dichroism spectra recorded at pH above 6 (Figs. S10, S13) also show the appearance of intense signals in the visible spectral region, which can be attributed to the coordination of the amide nitrogen (it is closer to the chiral α-carbon of the peptide backbone and therefore can produce a stronger CD absorption when the metal binding occurs). Increasing the pH value the intensity of CD bands increases suggesting the binding of a second amide that replaces a histidine in the equatorial plane of the complex, forming a (2N_Im_, 2N^–^) species and increasing the square-planar character of the corresponding complexes, [CuH_-2_L] (for **L1**) and [CuH_-1_L]^+^ (for **L2**)^25,26^. For Cu^2+^/**L2** system, increasing the pH two further species were detected [CuH_-2_L] and [CuH_-3_L]^–^. The former one is obtained with a p*K*_a_ = 9.44, likely corresponding to the deprotonation of the phenolic group of tyrosine, which does not participate in the metal complexation, while the latter one is formed under the most alkaline conditions and can be ascribed to the displacement of a third amide proton, giving rise to the (N_Im_, 3N^–^) species (*λ*_max_ = 525 nm) (Fig. 1a). The employed experimental techniques however do not allow to establish which His residues or backbone amides mainly participate in the complex formation.

As mentioned above, **L3** behaves differently than the acetylated analogues. Close to neutral pH, we observe the formation of the [CuH_3_L]^3+^ species, which dominates around pH 6.5. The Vis spectra show a clear blue-shift between pH 5.5 (where [CuH_4_L]^4+^ complex is predominant) and pH 6.5, but the wavelength of maximum absorption does not drop below 600 nm, as expected for 4N-type Cu^2+^ complexes (*i.e.* with four equatorially bound nitrogen atoms). Therefore, it can be assumed that the increase of pH favors the coordination of the terminal amine that replaces an imidazole donor group in the metal coordination sphere (Fig. 1b). The prevailing complex at physiological pH is [CuH_2_L]^2+^: the wavelength of maximum absorption significantly decreases, and the value recorded at pH 7.5 (553 nm) is very close to both those expected for a coordination (3N_Im_, N^–^) (557 nm) and (2N_Im_, NH_2_, N^–^) (549 nm). It cannot be excluded that both complexes are present in solution, or that it may exist a continuous and rapid “interconversion” between them, given the lability of the coordination bonds with the Cu^2+^ ion. Above pH 7.5, once again we observed the gradual coordination of three backbone amides, forming (2N_Im_, 2N^–^) and (N_Im_, 3N^–^) species, which dominate under alkaline conditions. The tyrosine and the two lysine residues simply lose their proton without participating in the complexation.

### Formation of zinc complexes with L1, L2 and L3

**Figure 2:**
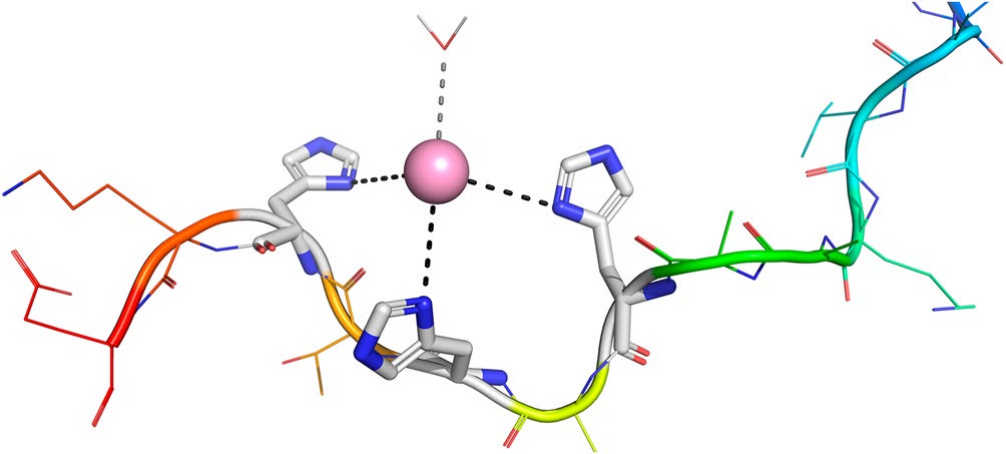
Coordination hypothesis for the (3N_Im_) Zn^2+^ complexes. Molecular structure generated with PyMol^27^.

Only mononuclear zinc complexes have been identified under the employed experimental conditions. MS spectra confirm the presence of different Zn^2+^ complexes with various protonation states: [ZnHL]^3+^ (**L1**, *m/z* = 599.3), [ZnL]^2+^ (**L2**, *m/z* = 898.4), [ZnH_3_L]^3+^ (**L3**, *m/z* = 584.6) and [ZnH_2_L ∙ K]^3+^ (**L3**, *m/z* = 597.3) (Table S4). For all the systems, the stoichiometry of the first identified species indicates that only one histidine residue is protonated and therefore the Zn^2+^ ion should coordinate two imidazole nitrogens, with a geometry that can be reasonably assumed to be tetrahedral^28^. The terminal Glu residue (or at least one of its carboxylate moieties in **L1**) can also bind the metal replacing a water molecule, as already reported for wild-type calcitermin^9^. When pH is increased, an acidic proton is released from the third histidine with a p*K*_a_ of 5.80 (**L1**), 6.14 (**L2**) and 5.6 (**L3**), forming the species (3N_Im_) where the third His can replace the carboxylate group (if coordinated) in the set of donor groups. A further deprotonation step leads to the formation of complexes [ZnH_-1_L]^+^ (**L1**, p*K*_a_ = 7.33), [ZnL]^2+^ (**L2**, p*K*_a_ = 7.63) and [ZnH_3_L]^3+^ (**L3**, p*K*_a_ = 7.32). In these cases, the release of the proton is attributable to the ionization of a coordinated water molecule. Increasing the pH, the complex-formation pattern and the geometry of the formed complexes with the acetylated peptides, **L1** and **L2**, are practically the same, with the only difference that, in the case of **L2**, we observe the deprotonation of the Tyr residue (p*K*_a_ = 9.44) without metal coordination. The formed zinc species with **L1** and **L2** are most likely tetrahedral complexes with two or three coordinated histidines (Fig. 2), while water molecules saturate the coordination sphere. Instead, in the case of **L3**, after the formation of [ZnH_3_L]^3+^, we observe the mere deprotonation of the four basic sites of the ligand, in the order: the terminal amine (p*K*_a_ = 7.69), the phenolic oxygen of tyrosine (p*K*_a_ = 9.47), and the two ε-amino groups of lysines. In the end, above pH 9 and in the case of the amidated peptides, the formation of [ZnHL]^+^ (for **L3**) and [ZnH_-1_L]^+^ (for **L2**) may be due to the release of an acidic proton from a second water molecule, which occupies a coordination position in a trigonal bipyramidal complex.

### Comparison of metal chelating abilities

Competition diagrams (Fig. S24) allow to compare the overall capacity of the studied ligands to coordinate Zn^2+^ or Cu^2+^ ions. Although they can be used only for a qualitative comparison of the binding affinities, the stability of the formed complexes in the entire explored pH range, regardless of stoichiometry and structure of the formed species, can be evaluated.

In Fig. S24a the affinity for copper of the wild-type calcitermin is compared to that of its protected derivatives. The metal chelating ability of **WT** and **L1** is the same under acidic conditions. The presence of the terminal carboxylic group moderately enhances the stability of the formed complexes. Above pH 4.5, **WT** exhibits the best copper binding ability. Both the free terminal amine and carboxylate contribute to thermodynamically favour the formed complexes, thanks to the presence of a higher number of donor sites. On the other hand, the metal binding ability of the protected analogues above pH 5 is comparable. This is in accordance with the speciation models that indicate a similar coordination behaviour for these systems, with the progressive binding of the three histidines, followed by the terminal amino group and the backbone amides.

In the case of zinc (Fig. S24b), **WT** is a slightly better ligand in the entire pH range. The protected analogues behave similarly up to pH 6, after which the greater stability is shown by the complexes formed by the *N-*acetylated peptide, **L1**. This trend can confirm that the terminal amine is not involved in zinc complexation. On the contrary, the *C-*terminal carboxyl group seems to stabilize the formation of the complexes; suggesting that Glu residue takes part in the coordination by means of its side chain and/or terminal COOH, as in the case of WT^9^.

### Peptide stability in plasma

**Figure 3:**
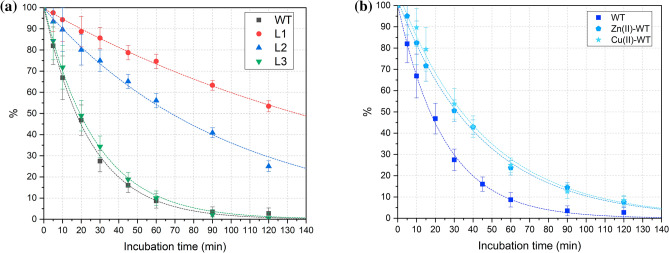
Stability in human plasma of wild-type calcitermin (**WT**) and its (**a**) *N-* and/or *C-*terminal protected analogues (**L1**, **L2**, **L3**); or (**b**) Zn^2+^ and Cu^2+^ complexes.

The stability of the investigated peptides in human plasma is reported in Fig. 3a and is compared with the proteolytic susceptibility of wild-type calcitermin (**WT**). The concentration of all the peptides decreases with an approximately exponential decay and a half-life period (t_1/2_) of about 20 min for **L3**, 68 min for **L2** and more than two hours (135 min predicted with an exponential regression) in the case of **L1**. The degradation profile of **L3** follows that of the native peptide, with a t_1/2_ comparable to the value obtained for **WT** (t_1/2_ = 18 ± 3 min). On the contrary the acetylated peptides, **L1** and **L2**, showed an improved resistance to degradation, with **L1** displaying the highest persistence in the human plasma. This result highlights the efficacy of the *N-*terminus protection as a general method to increase the resistance to proteolytic degradation of antimicrobial peptides^8^. The amidation of the *C-*terminus, instead, seems to affect less the resistance of calcitermin toward the activity of peptidases.

The effect of metal complex formation on the peptide stability has been evaluated for **WT** and **L1**—the least and most stable peptides, respectively. The complexation of Cu^2+^ and Zn^2+^ increases the half-life of **WT** from 18 min to about 33 min (Fig. 3b), while the t_1/2_ rises from 135 min to ≈150 min (Zn^2+^) or ≈160 min (Cu^2+^) in the case of **L1** (Fig. S25).

### Antimicrobial activity

The antimicrobial activity of **WT** calcitermin and its protected derivatives (**L1, L2, L3**) was assessed on *C. albicans*, *S. aureus*, and *E. coli*, respectively representative of human pathogens of fungal and bacterial origin.

**Figure 4:**
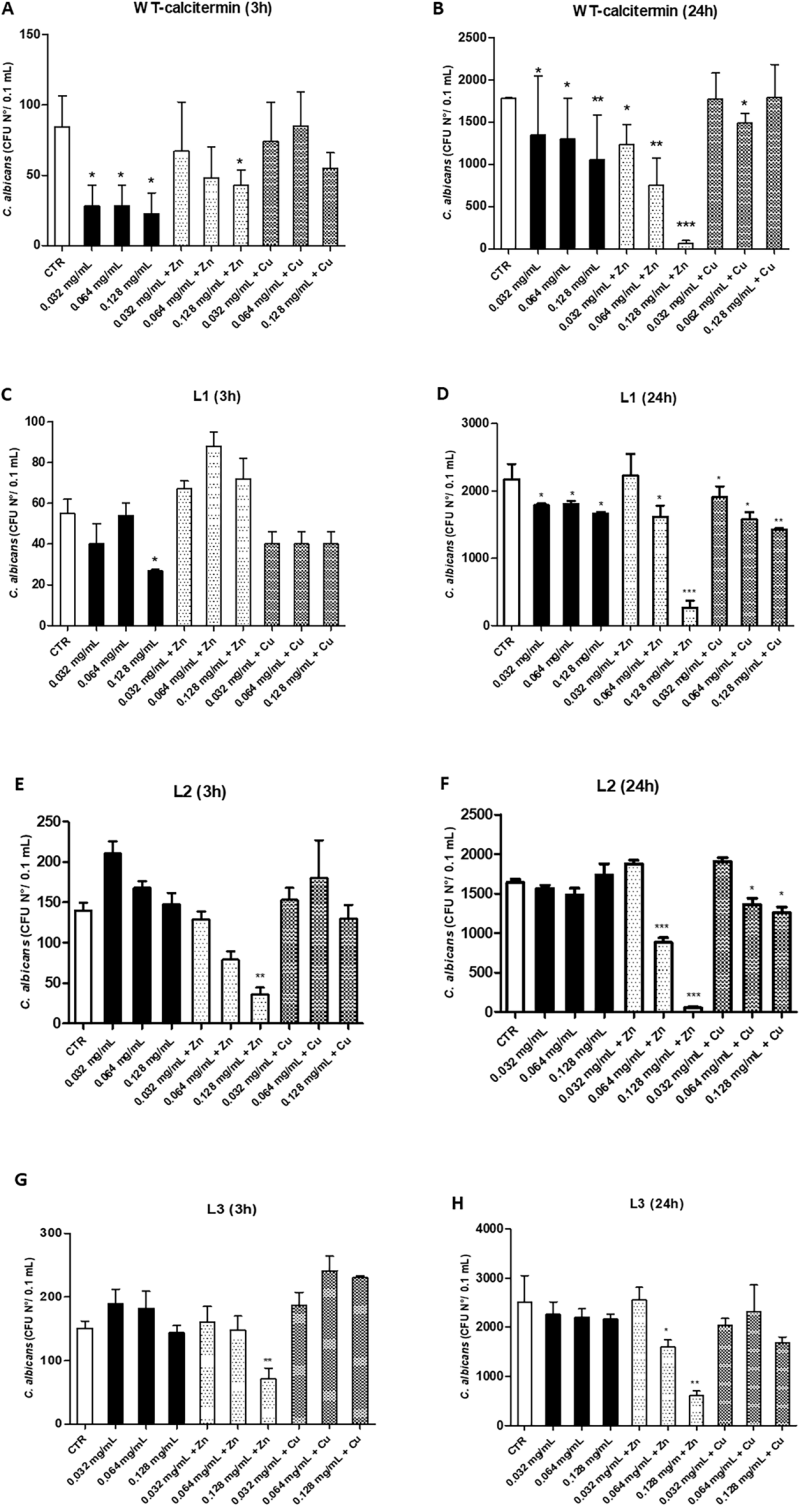
In vitro antifungal activity of **WT** and **L1, L2, L3** derivatives. All assays were performed in the presence or absence of ZnCl_2,_ or CuCl_2_ in aqueous buffer (5.4 pH). (**A**,**B**) WT-calcitermin action at 3 and 24 h-incubation; (**C**,**D**) L1 action at 3 and 24 h-incubation; (**E**,**F**) L2 action at 3 and 24 h-incubation; (**G**,**H**) L3 action at 3 and 24 h-incubation. Results are expressed ad mean value of CFUs ± SD obtained in triplicate samples from two independent experiments; *p ≤ 0.05; **p ≤ 0.01; ***p ≤ 0.001.

Regarding *C. albicans* (Fig. 4), the results showed a remarkable CFU reduction of the yeast in the presence of **WT** compared to untreated controls. The reduction was already evident after 3 h (Fig. 4A), with all used concentrations, despite a true dose-dependence was not observable (− 67%, − 67%, and − 73%, for 0.032–0.064 and 0.128 mg/mL respectively, *p* < 0.05). A significant reduction was also observed with 0.128 mg/mL **WT** in the presence of ZnCl_2_ (− 49%, *p* < 0.05), whereas no significant reduction was observed for the Cu^2+^/**WT** complex_._ After 24 h of contact, the reduction was maintained and remarkably increased by ZnCl_2_ presence, especially at 0.128 mg/mL (− 96%, *p* < 0.0001) (Fig. 4B). Instead, at 3 h, the **L1** derivative exhibited some antifungal action only at 0.128 mg/mL (Fig. 4C). At 24 h, limited yet significant CFU reduction was observed with **L1** alone (− 18%, − 17%, and − 23%, for 0.032, 0.064, and 0.128 mg/mL, respectively; *p* < 0.05), and increased activity was detected with the addition of ZnCl_2_ (− 26% and − 88%, for 0.064 mg/mL and 0.128 mg/mL, *p* < 0.05 and *p* < 0.001, respectively). Some CFU decrease was also observed with **L1** + CuCl_2_ (− 12%, − 28% and − 34% for 0.032, 0.064, and 0.128 mg/mL, respectively; *p* < 0.05) (Fig. 4C,D). Similarly, at 3 h, the **L2** derivative was significantly active only at 0.128 mg/mL in the presence of ZnCl_2_ (− 74%,* p* < 0.01), while at 24 h a significant CFU reduction was observed at 0.064 and 0.128 mg/mL plus ZnCl_2_ (respectively − 46%, *p* < 0.0001; and − 97%, *p* < 0.0001) and plus CuCl_2_ (− 17% and − 23%, respectively; *p* < 0.01) (Fig. 4E,F). Comparable results were observed for **L3**, which at 3 h was active only at 0.128 mg/mL in the presence of ZnCl_2_ (− 51%, *p* < 0.01), and at 24 h exhibited antifungal activity at 0.064 and 0.128 mg/mL in the presence of ZnCl_2_ (respectively − 37%, *p* < 0.05; and − 76%, *p* < 0.01) (Fig. 4G,H). ZnCl_2_ alone, included as a control, exhibited a significant antifungal activity in all performed assays, reducing *C. albicans* CFU number up to 64.9% at 3 h and to 94% at 24 h (*p* < 0.0001). By contrast, negligible or no CFU reduction was observed in all conditions with CuCl_2_ alone, which significantly reduced *C. albicans* CFU number only at 24 h, in one out of the total performed assays (Table S5).

Summarizing, the results on *C. albicans* confirmed the antifungal activity of **WT** calcitermin, even at low concentrations, at both 3 and 24 h. The **WT**/zinc complex was quite effective against *C. albicans* at 24 h, at 0.128 mg/mL, although only a slightly lower activity was found for ZnCl_2_ itself at the same concentration. On the other hand, no activity was shown by CuCl_2_ alone and its presence did not significantly affect the **WT** activity, although Cu^2+^ can form stable complexes with calcitermin even at pH 5.4. Of note, previous studies showed that both Zn^2+^ and Cu^2+^ ions possess antifungal activity per se at concentrations consistent with those used in our study, showing similar minimal inhibitory concentrations, although Zn^2+^ was generally proven to be more effective compared to Cu^2+^^29–31^. Our results confirmed the Zn^2+^ anti-*C. albicans* activity, whereas a marked antifungal activity by Cu^2+^ ion was not evidenced. This might be due to: the different type of assay performed in our study compared to those previously reported; the different metal ion formulations tested (Zn/Cu chloride vs. Zn/Cu oxide), the absence in our study of a nanoparticle delivery system^32,33^; the suboptimal concentrations of ions used in our assays, which may have induced some yeast tolerance to metal ions. Further studies would be needed to address these points. The amidation of the carboxyl terminal (peptides **L2** and **L3**) did not improve the antifungal activity of **WT**, also in the presence of zinc. Some effect due to acetylation of the *N-*terminus of calcitermin (**L1** and **L2** peptides) was observed in the presence of CuCl_2_, after 24 h; in particular, Cu^2+^ complexes with a concentration of 0.128 mg/mL were significantly more active than both the corresponding controls and the calcitermin complexes.

**Figure 5:**
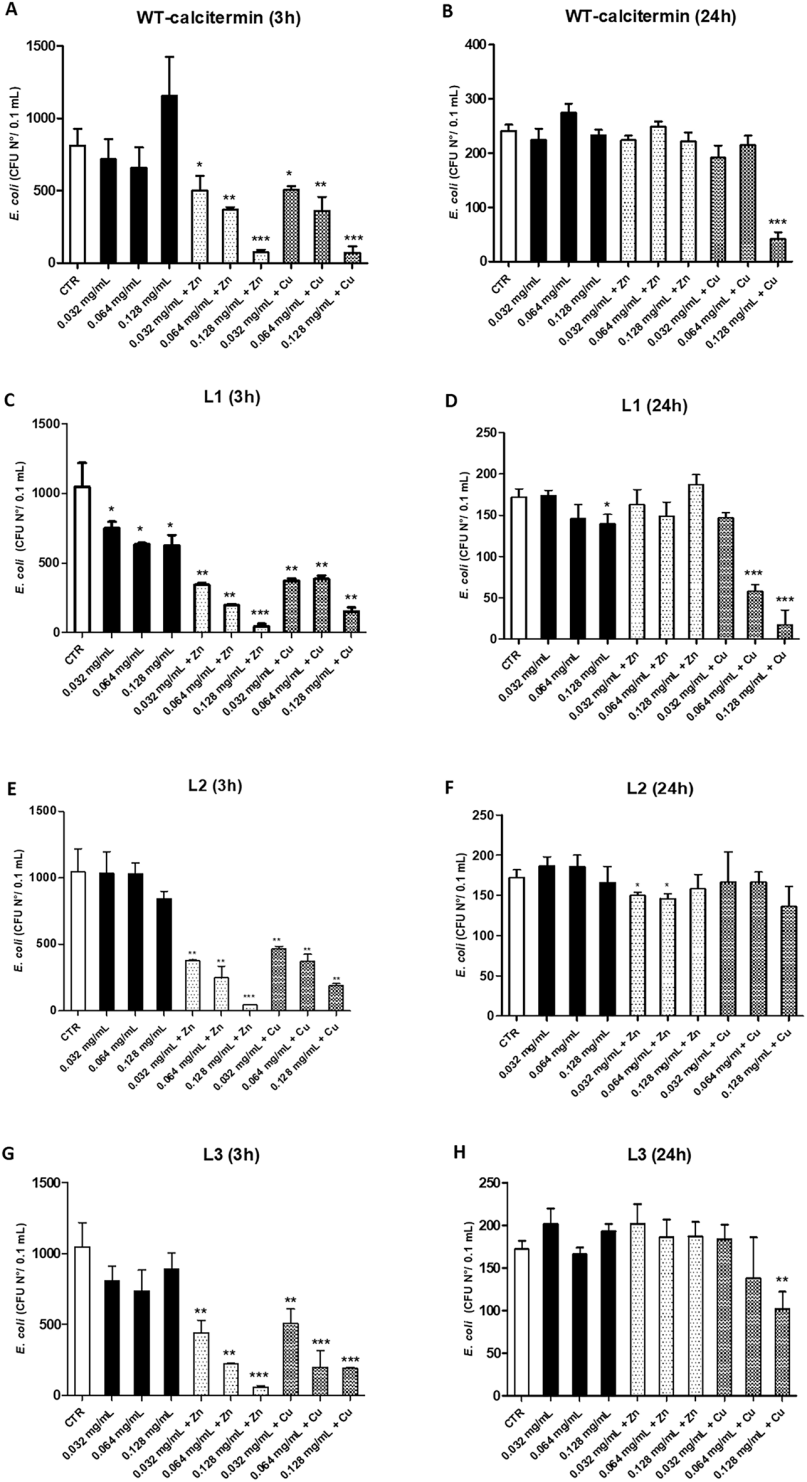
In vitro anti-*E. coli* activity of **WT** and **L1, L2, L3** derivatives. All assays were performed in the presence or absence of ZnCl_2,_ or CuCl_2_ in aqueous buffer (5.4 pH). (**A**,**B**) WT-calcitermin action at 3 and 24 h-incubation; (**C**,**D**) L1 action at 3 and 24 h-incubation; (**E**,**F**) L2 action at 3 and 24 h-incubation; (**G**,**H**) L3 action at 3 and 24 h-incubation. Results are expressed ad mean value of CFUs ± SD obtained in triplicate samples from two independent experiments; *p ≤ 0.05; **p ≤ 0.01; ***p ≤ 0.001.

The antimicrobial action of calcitermin and its derivatives was also tested on bacteria, including the Gram-negative bacillus *E. coli* and the Gram-positive coccus *S. aureus*. Regarding *E. coli,* the results showed that **WT** was active essentially in the presence of ZnCl_2_ or CuCl_2_ (Fig. 5A,B). At 3 h, the CFU number decreased to − 38% (*p* < 0.05), − 55% (*p* < 0.01), and − 91% (*p* < 0.001) with respect to controls, using 0.032, 0.064, and 0.128 mg/mL of peptide + ZnCl_2_, respectively. With the addition of CuCl_2_, the CFU decrease corresponded to − 38% (*p* < 0.01), − 56% (*p* < 0.05), and − 91% (*p* < 0.001) for 0.032, 0.064, and 0.128 mg/mL of peptide, respectively. At 24 h, no significant CFU reduction was observed with **WT** with or without ZnCl_2_, whereas a − 82% decrease of CFU number was detected with 0.128 mg/mL of calcitermin in the presence of CuCl_2_ (*p* < 0.0001).

Differently from **WT**, the **L1** derivative induced a dose-dependent CFU decrease at 3 h (− 28%, − 39%, and − 40%, for 0.032, 0.064, and 0.128 mg/mL, respectively; *p* < 0.05). The reduction was increased in the presence of ZnCl_2_ (− 67%, − 81%, and − 96%, respectively; *p* < 0.001), and CuCl_2_ (− 65%, − 63%, and − 85% for 0.032, 0.064, and 0.128 mg/mL, respectively; *p* < 0.01). At 24 h, **L1** was active only at 0.128 mg/mL, alone (− 19%, *p* < 0.05) or in the presence of CuCl_2_, which significantly increased **L1** action (− 67% and − 90% for 0.064 and 0.128 mg/mL, respectively; *p* < 0.001) (Fig. 5C,D).

A similar trend was observed for **L2**, which at 3 h reduced *E. coli* CFUs only in the presence of ZnCl_2_ (− 64%, − 76%, and − 96% at 0.032, 0.064 and 0.128 mg/mL, respectively; *p* < 0.001) or CuCl_2_ (− 55%, − 65%, and − 82%, respectively; p < 0.01). At 24 h, no significant CFU reduction was observed except for 0.032 and 0.064 mg/mL in the presence of ZnCl_2_ (Fig. 5E,F). The activity of the **L3** derivative was essentially detectable only at 3 h in the presence of ZnCl_2_ and CuCl_2_, inducing a significant CFU decrease at 0.032, 0.064 and 0.128 mg/mL (for ZnCl_2_, − 58%, − 79%, and − 94%, respectively, *p* < 0.001; for CuCl_2_, − 51%, − 81%, and − 82%, respectively, *p* < 0.001). After 24 h, no significant reduction was observed at any L3 concentration, except for 0.128 mg/mL in the presence of CuCl_2_ (− 41%, *p* < 0.01) (Fig. 5G,H).

As also observed in the antifungal assays, ZnCl_2_ exhibited an anti-*E. coli* activity per se, reducing the CFU number up to − 95% at 3 h (*p* < 0.0001), although it was not anymore active at 24 h, showing a − 30% reduction only in one of the performed assays (p < 0.05). Also CuCl_2_ displayed a high antibacterial activity, inducing an up to 96% reduction at both 3 and 24 h of incubation (*p* < 0.0001) (Table S6). In conclusion, only the derivative **L1** showed a significant anti-*E. coli* activity in the absence of metals, especially after 3 h. Interestingly, **L1** was also the least sensitive peptide of the series towards the proteolytic enzymes. Copper and zinc exhibited antimicrobial activity against *E. coli* at 3 h, both in their free and complexed form; this behaviour was very similar for all peptides, regardless of the protection of the terminals. However, at 24 h, only copper (and its complexes) showed some activity, especially in the case of **L1**, whose complexes with Cu^2+^ were already active at the concentration of 0.064 mg/ml.

**Figure 6:**
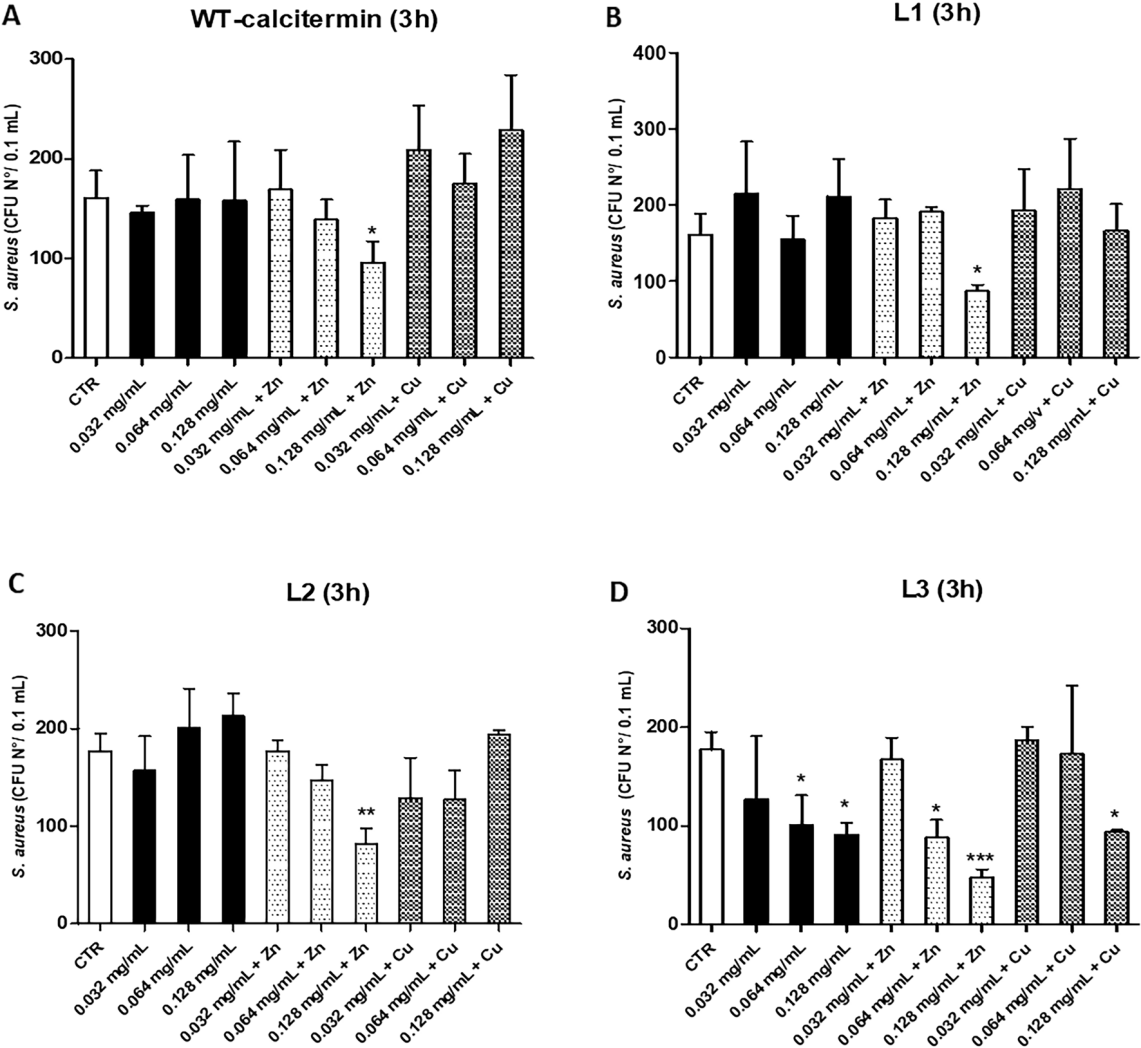
In vitro anti-*S. aureus* activity of **WT** and **L1, L2, L3** derivatives. All assays were performed for 3 h-incubation time in the presence or absence of ZnCl_2,_ or CuCl_2_ in aqueous buffer (pH 5.4). (**A**) WT-calcitermin action; (**B**) L1 action; (**C**) L2 action; (**D**) L3 action. Results are expressed ad mean value of CFUs ± SD obtained in triplicate samples from two independent experiments; *p ≤ 0.05; **p ≤ 0.01; ***p ≤ 0.001.

Regarding *S. aureus*, the assays were performed only at 3 h, since at 24 h of incubation in aqueous buffer at pH 5.4 no viable bacteria were detectable even in untreated controls. At 3 h, the data showed that **WT** was significantly active only at 0.128 mg/mL in the presence of ZnCl_2_ (− 41%, *p* < 0.05) (Fig. 6A).

Similarly, both **L1** and **L2** derivatives induced a significant CFU decrease only at 0.128 mg/mL in presence of ZnCl_2_ (− 46%, *p* < 0.05; − 54%, *p* < 0.01, respectively for **L1** and **L2**) (Fig. 6B,C). **L3** action was instead more evident, inducing a CFU decrease corresponding to -28% (*p* = n.s.), − 43% (*p* < 0.05), and − 49% (*p* < 0.05) at 0.032, 0.064, and 0.128 mg/mL concentration, respectively. Moreover, the action of 0.128 mg/mL of **L3** was increased by the addition of ZnCl_2_ (− 73% CFU decrease, *p* < 0.001) (Fig. 6D), and by the presence of CuCl_2_ (− 47%, *p* < 0.01) (Fig. 6).

Likewise, ZnCl_2_ alone showed a clear antimicrobial activity toward *S. aureus*, inducing an up to 77% CFU reduction, compared to controls (Table S7). Regarding *S. aureus*, in conclusion, **WT** calcitermin showed no effect, but a significant antimicrobial activity was observed for **L3**. Zn^2+^ complexes of all peptides were active at higher concentrations, especially when the terminals were protected. Among the Cu^2+^ complexes, only the one with **L3** showed a detectable action, at a concentration of 0.128 mg/ml, although this was comparable to that shown by the same peptide in the absence of metals.

## Concluding remarks

Although calcitermin and its antimicrobial activity have been known for over twenty years, this peptide is still sparsely studied as a potential new drug. The studies of Cole and coworkers^10^ first showed that bivalent endogenous metals, such as zinc and copper, can modulate the antimicrobial activity of calcitermin; later on, our research group investigated its metal binding properties. Then, a systematic study on calcitermin derivatives has begun, modifying the amino acid sequence in an opportune manner, with a view to improving interaction with cell membranes, to increasing stability towards proteolytic enzymes and/or to improving the sequestering ability towards metal ions to promote nutritional immunity. The encouraging previous results have led us to study the new calcitermin derivatives reported in this paper, where the native peptide has been protected at one or both of its termini. This is one of the ways, already known in the literature, to increase the resistance to proteolytic degradation of a peptide^34–37^. However, since any variation in the structure of the peptide can affect both its ability to form metal complexes and its antimicrobial properties, we have carried out a thorough study, both from the chemical and microbiological point of view, to shed light on the behaviour of the modified peptides. The formation of metal complexes with calcitermin is driven by the presence of its three histidine residues. This coordination site is preserved in all peptides studied here, and stable complexes with all ligands have always been observed. According to Pearson’s classification, in fact, the imidazole side chain of histidine is a good binding site (borderline basic character) for Cu^2+^ and Zn^2+^ ions (borderline Lewis acids)^38^ and, on the basis of their electronic configuration, the Irving-Williams series also predicts a higher binding affinity for Cu^2+^ than Zn^2+^^39^.

The metal coordination did not affect the peptide structure, and for all the systems a prevalence of random coil conformation was observed (Figs. S16–S18). However, the contribution to the complex-formation by the amino and carboxyl termini, although secondary, is evident: both with copper and zinc, the most stable complexes are always those formed by the native calcitermin, throughout the explored pH range. Noteworthy, the metal complexed forms of the studied peptides display higher resistance to degradation. Indeed, the formation of both copper and zinc complexes with calcitermin almost doubled its half-life in human plasma, suggesting that the interaction with metal ions can be a fruitful strategy to increase the peptide stability in biological fluids. Whether this correlates with the slightly better antimicrobial activity of the studied metal complexes is not easy to judge, and further investigation is required. Similarly, it is not straightforward to link the antimicrobial activity of the studied peptides with their stability in plasma.

Acetylation of the *N-*terminal confers a much longer half-life with respect to peptides with free amino terminus. This is an important indication for the design of calcitermin derivatives (as well as other AMP derivatives)^35–37^ that may persist in the body long enough to perform their microbicidal action. Indeed, the study of peptide degradation will be deepened trying to identify the responsible enzymes and the most vulnerable peptide bonds, also extending the investigation to other biological fluids, such as saliva and gastric juices. Moreover, a preliminary investigation on peptide interaction with albumin (one of the major proteins in plasma) has been carried out to evaluate the protein-peptide binding, which may affect the decrease of peptide concentration detected after incubation in plasma. We observed a variation in the α-helical content of albumin structure when the studied peptides are added to the solution (Fig. S26). The mechanism and effect of such interaction will require further specific investigations; however, all the investigated peptides, even in their metal complexed form, behave in the same way and decrease the same extent the α-helix structure of the protein. Therefore, the interaction with albumin does not affect the relative stability of calcitermin and its derivative systems.

Lastly, although the new calcitermin derivatives studied here were not particularly promising as commercial antimicrobials against fungi such as *C. albicans* or against Gram-positive or Gram-negative bacteria, encouraging results have been obtained for their metal (especially Zn^2+^) complexes. The investigation will be therefore extended to other derivatives, other microorganisms and other experimental conditions. In conclusion, calcitermin and its derivatives remain a promising class of peptides of great interest as new generation of metal-enhanced antimicrobials.

## Methods

### Potentiometry

Stability constants for proton and metal complexes were obtained from pH-metric titration curves registered at *T* = 298 K and ionic strength 0.1 M (KCl). The potentiometric apparatus was previously described^40^. Solutions were titrated with 0.1 M carbonate-free KOH. The electrode was daily calibrated for hydrogen ion concentration by titrating HNO_3_ with the standard base solution, under the same experimental conditions as above. The asymmetry potential and the slope of the electrode couple were computed by means of SUPERQUAD^41^ and Glee^42^ programs. The purities and exact concentrations of the ligand solutions were determined by the Gran method^43^. The HYPERQUAD^44^ program was employed for the overall formation constant (*β*) calculations, referred to the following equilibrium equation: pM + qL + rH ⇆ M_p_L_q_H_r_ (charges omitted; p is 0 in the case of ligand protonation; r can be negative). The computed standard deviations (referring to random errors only) were given by the program itself and are shown in parentheses as uncertainties on the last significant figure. Hydrolysis constants for metal ions were taken from the literature^45,46^. The distribution and competition diagrams were computed using the HYSS program^47^.

### Mass spectrometry

High-resolution mass spectra were obtained on the LCMS-9030 qTOF Shimadzu (Shimadzu, Kyoto, Japan), equipped with a standard ESI source and the Nexera X2 system. The instrumental parameters were as follows: positive ion mode, scan range *m*/*z* 100–3000, dry gas nitrogen, temperature 170 °C, and ion energy 5 eV. The capillary voltage was optimized to the highest S/N ratio and it was 4500 V. The small changes in voltage (± 500 V) did not significantly affect the optimized spectra. The samples ([L]_tot_ = 1∙10^−4^ M and M:L molar ratio = 1:1) were prepared in a 1:1 methanol–water mixture at pH 5.2 and 7.4. They were directly infused at a flow rate of 3 μl/min. The instrument was externally calibrated with a Tunemix™ mixture (Bruker Daltonik, Germany) in quadratic regression mode. Data were processed using the Bruker Compass DataAnalysis 4.2 program. The mass accuracy for the calibration was > 5 ppm, enabling together with the true isotopic pattern (using SigmaFit) an unambiguous confirmation of the elemental composition of the obtained complex.

### Spectroscopic measurements

The absorption spectra of Cu^2+^ containing solutions were recorded on a Varian Cary50 Probe spectrophotometer, in the range 350–900 nm, using a quartz cuvette with an optical path of 1 cm. To describe the species present in solution, the observed wavelength of maximum absorption at a given pH was compared with the expected *λ*_max_ value obtained from literature^23,48–50^. Circular dichroism (CD) spectra were recorded on a Jasco J-1500 spectropolarimeter in the 180–800 nm range, using a quartz cuvette with an optical path of 1 cm in the visible and near-UV range, and 0.01 cm in the 180–250 nm range. Electron paramagnetic resonance (EPR) spectra were recorded in liquid nitrogen on a Bruker ELEXSYS E500 CW-EPR spectrometer at X-band frequency (9.5 GHz) and equipped with an ER 036TM NMR teslameter and an E41 FC frequency counter. Ethylene glycol (30%) was used as a cryoprotectant. The EPR parameters were analyzed by computer simulation of the experimental spectra using WIN-EPR SIMFONIA software, version 1.2 (Bruker, Billerica, MA, USA).

### Peptide stability in plasma

The persistence of calcitermin and its analogues and metal complexes in human plasma has been studied in vitro by means of the following procedure^51^. Each peptide (C = 1·10^–4^ M) and metal complex (metal:ligand ratio 1:1) was incubated in a sample containing 1 ml of human plasma (obtained as a pool of 40 individuals from the University Hospital of Ferrara) and 1 ml of ammonium acetate buffer (pH 7.4) at 37 °C. L-phenylalaninol is added as internal standard. At regular time intervals, 200 μl aliquots were taken and enzymatic reaction was blocked by adding 300 μl of HClO_4_ 0.5 M. The sample was then centrifugated for 6 min at 14,000 × *g*. The supernatant was then filtered and analysed by HPLC. The analytical column was an Agilent Poroshell 120 SB-C18 (4.6 × 100 mm, 2.7 μm, pore size 120 Å). Flow rate = 0.5 ml/min, mobile phase H_2_O + 0.1% v/v TFA, linear gradient from 0 to 60% of acetonitrile + 0.1% v/v TFA over 30 min for the elution of peptides, temperature 25 °C, detection wavelength 210 nm. The results on calcitermin are the mean of three independent measurements while only one test was performed with the three protected peptides; in the latter cases, the errors have been estimated on the basis of those obtained for the wild-type calcitermin. Informed consent was obtained from all subjects and/or their legal guardian(s) prior to the donation of blood samples. All experimental protocols were approved by the National Research and Research Transversal Processes boards at University of Ferrara. All methods were carried out in accordance with relevant guidelines and regulations.

### Biological tests

The potential antimicrobial activity of wild-type calcitermin and *N-* and *C-* terminally protected derivatives was investigated by using the yeast *C. albicans* (ATCC 10231), the Gram-positive *S. aureus* (ATCC 25293), and the Gram-negative *E. coli* (ATCC 25922) (all from American Type Culture Collection, ATCC, Thermo Scientific, Milan, Italy). All microbes were grown at 37 °C in Tryptic Soy Broth (TSB; Biolife, Milan, Italy) or Tryptic Soy Agar plates (TSA, Biolife, Milan, Italy). Peptides were tested at a final concentration C = 0.128, 0.064, 0.032 mg/mL, in the presence or absence of ZnCl_2_ or CuCl_2_, at a M:L molar ratio 0.9:1. Briefly, all the microbes were expanded in TSB at 37 °C under agitation for 12 h and then sub-cultured by 1:10 dilution in TSB under the same conditions until reaching OD_600nm_ = 0.3–0.4, as measured by spectrophotometric reading using a GENESYS 10S UV–Vis spectrophotometer (Thermo Scientific, Milan, Italy), corresponding to 0.9–1.2 × 10^7^ colony forming units (CFU) per mL, 1.5–2 × 10^8^ CFU/mL and 2.4–3.2 × 10^8^ CFU/mL, for *C. albicans, S. aureus* and *E. coli* respectively. The cultures were then centrifuged at 4500 rpm for 10 min in a SL 8 centrifuge (TX-150 Rotor) (Thermo Scientific, Milan, Italy), and the cellular pellets were washed with PBS pH 5.4. Pelletized cultures were then suspended in 10 mL of PBS pH 5.4, then the suspensions were diluted to a final concentration of 10^4^ CFU/mL. Aliquots of 100 µl (corresponding to 10^3^ CFU) were seeded in each well of a 96-wells plate. 3 µl of TSB at pH 5.4 were added to each well to obtain a final 1.5% TSB concentration in the culture medium. Peptides were serially diluted in PBS pH 5.4 to C = 0.257, 0.128, and 0.064 mg/mL. ZnCl_2_ and CuCl_2_ solutions were added where requested to obtain a M:L ratio 0.9:1. 100 µl of the prepared peptides ± ZnCl_2_/CuCl_2_ solutions were then added to each well, obtaining the final 0.128, 0.064 and 0.032 mg/mL peptide concentrations in a total 0.2 mL volume. PBS alone and PBS + ZnCl_2_/CuCl_2_ solutions were included as controls. The plates were incubated at 37 °C on a platform rocker with slow stirring for 3 and 24 h. At the end of the incubation times, 100 µl aliquots were taken from each sample and diluted in PBS pH 5.4 to be seeded on TSA plates. Specifically, aliquots were diluted 1:10 at 3 h, and 1:200, 1:100,000 and 1:10 at 24 h, for *C. albicans*, *E. coli*, and *S. aureus* respectively. After incubation for 24 h at 37 °C, grown CFUs were enumerated. Samples were assayed in triplicate in two independent experiments. Student’s* t*-test was used for the evaluation of significance in biological tests; a *p* value ≤ 0.05 was considered significant.

### Supplementary Information

Supplementary Information.

## Data Availability

The datasets used in the current study is available from the corresponding author upon request.
