# Supplementary material for: Impact of C- and N-terminal protection on the stability, metal chelation and antimicrobial properties of calcitermin
Source: Sci Rep. 2023 Oct 25;13:18228. doi: 10.1038/s41598-023-45437-0 (PMC10600247; doi:10.1038/s41598-023-45437-0)
Supplement: Supplementary file 1 — Supplementary Information. [file 41598_2023_45437_MOESM1_ESM.pdf]

Impact of *C*- and *N*-terminal protection on the stability, metal chelation and antimicrobial properties of calcitermin

Maria D'Accolti,[1] Denise Bellotti,[1,2,*] Emilia Dzień,[2] Carlotta Leonetti,[1] Silvia Leveraro,[1] Valentina Albanese,[1] Erika Marzola,[1] Remo Guerrini,[1] Elisabetta Caselli,[1] Magdalena Rowińska-Żyrek [2] and Maurizio Remelli [1]

[1] *Department of Chemical, Pharmaceutical and Agricultural Sciences, University of Ferrara, Via L. Borsari 46, 44121, Ferrara, Italy*

[2] *Faculty of Chemistry, University of Wrocław, F. Joliot-Curie 14, 50-383, Wrocław, Poland*

* *Corresponding author*

Supplementary Information

Table of contents

1. Wild-type calcitermin: thermodynamic data
2. Protonation of N- and C-terminal protected derivatives of calcitermin
3. Speciation diagrams of metal complexes
4. Spectroscopic data: UV-Vis, CD and EPR
5. Far-UV circular dichroism
6. Mass spectrometry
7. Competition plots
8. Antimicrobial activity
9. Peptide stability in human plasma
10. CD assay for albumin-peptide systems
11. Materials
12. References

1. Wild-type calcitermin: thermodynamic data

Wild-type calcitermin (**WT**) has been previously investigated by our research group in aqueous solutions and ionic medium $NaClO_4$.[1] For the sake of comparison, potentiometric studies of **WT** and its copper and zinc complexes have been performed in a different ionic medium (KCl 0.1 M). The study of the speciation under different experimental conditions, changing ionic medium and metal-ligand ratio, is essential to comprehensively describe the thermodynamics of calcitermin, which is usually found in multicomponent media like biological fluids. The different ionic medium entails a slight difference in the obtained log*β* (Table S1), but without upsetting the previously proposed speciation model for **WT**. The potentiometric results confirm the coordination modes identified for copper and zinc complexes at different pH values. In the case of copper, we can ascertain the binding of three histidine residues under acidic conditions, the metal interaction with the terminal amine and, above pH 7, the participation in the coordination of up to three backbone amides. For zinc, we observe the formation of eight mononuclear species where the metal coordination sphere includes the three histidine residues, in accordance with literature.[1]

Table S1. Equilibrium constants for protonation of **WT** and its Cu^{2+} and Zn^{2+} complex formation at *T* = 298 K and *I* = 0.1 M (KCl). Values in parentheses are standard deviations on the last significant figure.

VAIALKAAHYHTHKE (WT)		
**Species**	**log*β***	**log*K***
HL^{2-}	10.61(6)	10.61
H_2L^{-}	21.14(2)	10.53
H_3L	30.64(4)	9.50
H_4L^{+}	38.38(4)	7.73
H_5L^{2+}	45.31(4)	6.93
H_6L^{3+}	51.64(4)	6.33
H_7L^{4+}	57.29(4)	5.65
H_8L^{5+}	61.51(4)	4.22
H_9L^{6+}	64.44(5)	2.93
$[CuH_6L]^{5+}$	56.40(6)	-
$[CuH_5L]^{4+}$	52.07(3)	4.34
$[CuH_4L]^{3+}$	46.97(3)	5.09
$[CuH_3L]^{2+}$	40.77(4)	6.21
$[CuH_2L]^{+}$	33.77(4)	7.00
$[CuHL]$	26.20(4)	7.58
$[CuL]^{-}$	16.83(5)	9.37
$[CuH_{-1}L]^{2-}$	6.90(5)	9.93
$[CuH_{-3}L]^{4-}$	-14.19(5)	-
$[ZnH_5L]^{4+}$	49.30(8)	-
$[ZnH_4L]^{3+}$	43.55(3)	5.75
$[ZnH_3L]^{2+}$	36.47(5)	7.08
$[ZnH_2L]^{+}$	28.59(5)	7.87
$[ZnHL]$	20.11(6)	8.48
$[ZnL]^{-}$	10.35(8)	9.77
$[ZnH_{-1}L]^{2-}$	-0.2(1)	10.5
$[ZnH_{-2}L]^{3-}$	-10.8(1)	10.7

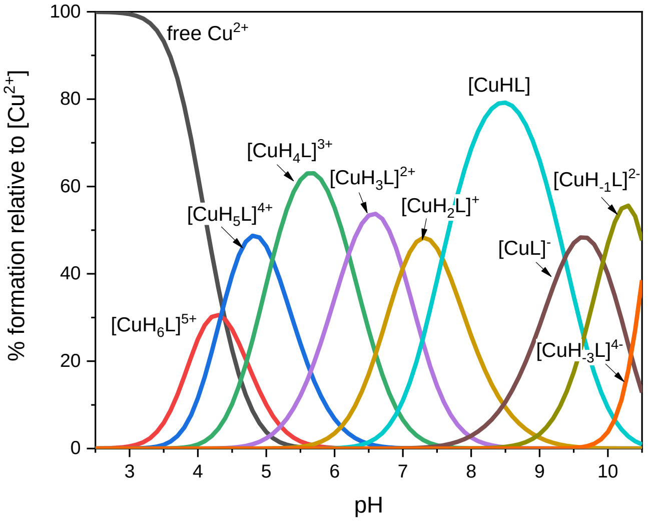

Figure S1. Species distribution diagram of Cu^{2+}/ **WT**; M:L ratio 0.9:1.

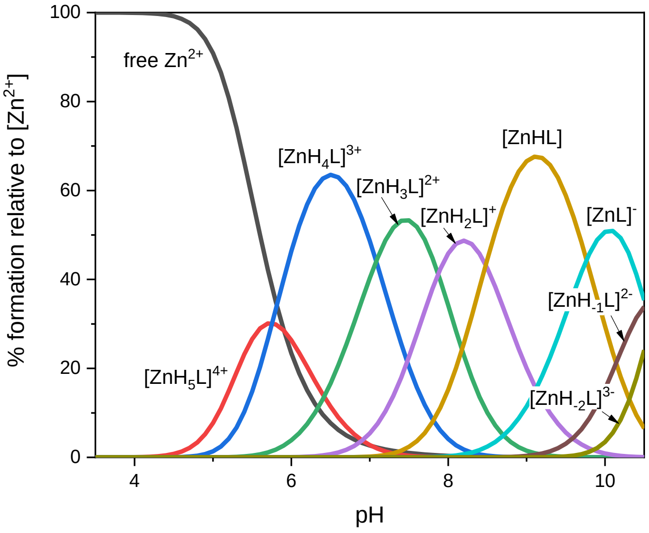

Figure S2. Species distribution diagram of Zn^{2+}/ **WT**; M:L ratio 0.9:1.

2. Protonation of N- and C-terminal protected derivatives of calcitermin

For each peptide, the obtained protonation *macro*-constants reported in Table S2 have been compared with literature data in order to assign the protonation step to the corresponding functional group. The symbol L here indicates the ligand in its unprotonated form where all the dissociable protons in the explored pH range have been released.

The Ac-VAIALKAAHYHTHKE (**L1**) peptide has the N-terminal end modified through acetylation, while the C-terminal end is free. The formation of a precipitate is observed at pH 8, where the neutral species is formed; in fact, at this pH, two positive charges come from the two Lys residues, which are still protonated, and two negative charges derive from the carboxylate groups of the C-terminus and the Glu residue. In the explored pH range (2.5–8.0) the deprotonations of tyrosine and lysine residues are hardly detectable, since they occur at higher pH values, and we therefore considered only five protonable sites in the peptide: three histidines, the glutamic acid and the terminal carboxylic group. The net charge of L, in this case, is zero.

Similarly, in the solution of Ac-VAIALKAAHYHTHKE-NH_2 (**L2**) peptide, in which both the termini are protected, precipitation occurred at pH 9.5. Once again, we can explain this precipitation with the formation of the neutral species which is predominant at this pH. Therefore, peptide **L2** has only five sites involved in acid-base reactions in the accessible pH range: three histidines, the glutamic acid and the phenolic group of tyrosine. Once again, the net charge of L is zero.

VAIALKAAHYHTHKE-NH_2 (**L3**) peptide has only the C-terminal group protected by amidation, while the free terminal amine can participate in acid-base reactions. Contrarily to the previous peptides, **L3** remains soluble throughout all the glass-electrode accessible pH range (2.5–10.5). Eight different species have been detected in solution, derived from the protonation of the following sites: three histidines, the glutamic acid, the phenolic group of tyrosine, two lysine ε-amino groups and the terminal amine. The unprotonated form of the peptide **L3** has therefore a net charge of -2 (L^{2-}).

The experimental protonation constants (Table S2) are in good agreement with the literature expectations.[2] The terminal carboxylic group, which is present only in **L1**, is the most acidic moiety with a log*K* value of 2.87. It is followed by the glutamic acid with a log*K* varying from 3.83 to 4.19. The protonation constants for His residues ranged instead from 5.55 to 7.17, in good agreement with the expectations based on similar systems.[2] Only ligand **L3** contains the free terminal amino group, which shows a log*K* = 7.72. Under alkaline conditions, the hydroxyl proton of tyrosine can be spontaneously released with p*K* values of 9.30 in the case **L2** and 9.53 for the terminally amidated analogue (**L1)**. The deprotonation constants of Lys residues have been measured only in the case of ligand **L3**, the only one of the three peptides which was possible to investigate until high pH values. The protonation of the two lysil ε-amino groups occurred with log*K* 10.19 and 10.76, respectively. Lastly, the deprotonation of the backbone amidic groups is not considered, since they are very weak acids ($pK_a \approx 15$)[3] and their spontaneous ionization occurs above the pH range explored for all the systems.

Table S2. Overall (*β*) and step (*K*) protonation constants for the investigated ligands (**L1**, **L2** and **L3**) at *T*=298 K and *I*=0.1 M (KCl). Standard deviations on the last significant figure are reported in parentheses.

	Ac-VAIALKAAHYHTHKE (**L1**)			Ac-VAIALKAAHYHTHKE-NH_2 (**L2**)		
**Species**	**log*β***	**log*K***	**Group/Residue**	**log*β***	**log*K***	**Group/Residue**
**HL^+**	7.10(1)	7.10	His	9.30(3)	9.30	Tyr
**H_2L^{2+}**	13.45(1)	6.35	His	16.44(5)	7.14	His
**H_3L^{3+}**	19.10(1)	5.65	His	22.58(5)	6.14	His
**H_4L^{4+}**	23.29(1)	4.19	Glu	28.32(5)	5.74	His
**H_5L^{5+}**	26.15(1)	2.87	C-term	32.31(5)	4.00	Glu

	VAIALKAAHYHTHKE-NH_2 (**L3**)		
**Species**	**log*β***	**log*K***	**Group/Residue**
**HL^-**	10.76(7)	10.76	Lys
**H_2L**	20.95(5)	10.19	Lys
**H_3L^+**	30.48(6)	9.53	Tyr
**H_4L^{2+}**	38.20(6)	7.72	N-term
**H_5L^{3+}**	44.99(6)	6.79	His
**H_6L^{4+}**	51.19(6)	6.20	His
**H_7L^{5+}**	56.73(6)	5.55	His
**H_8L^{6+}**	60.57(6)	3.83	Glu

3. Speciation diagrams of metal complexes

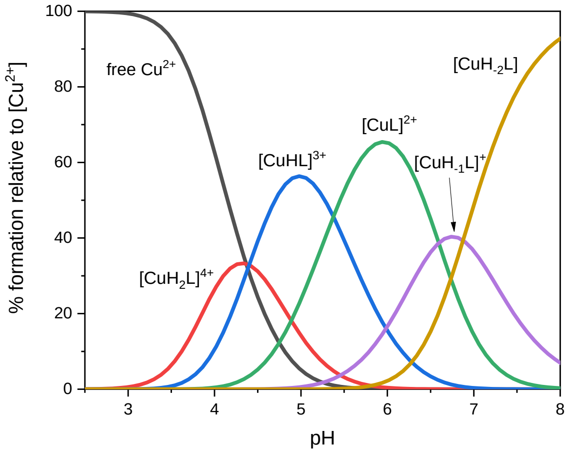

Figure S3. Species distribution diagram of Cu^{2+}/ **L1**; M:L ratio 0.9:1.

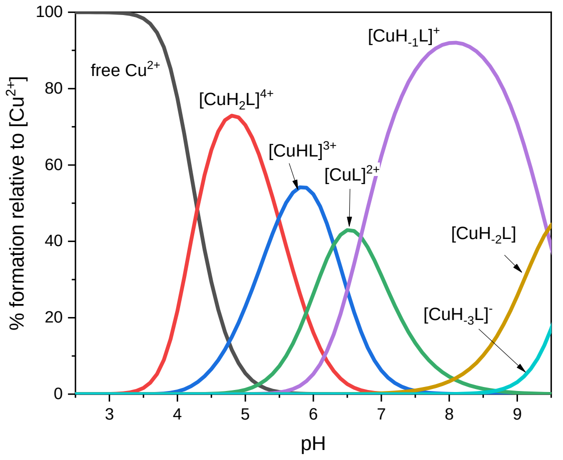

Figure S4. Species distribution diagram of Cu^{2+}/ **L2**; M:L ratio 0.9:1.

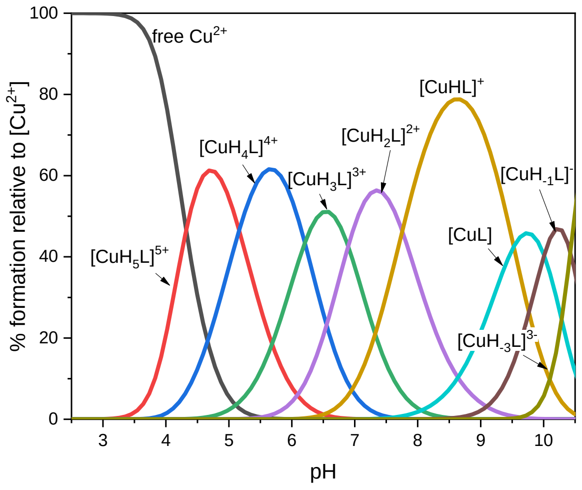

Figure S5. Species distribution diagram of Cu^{2+}/ **L3**; M:L ratio 0.9:1.

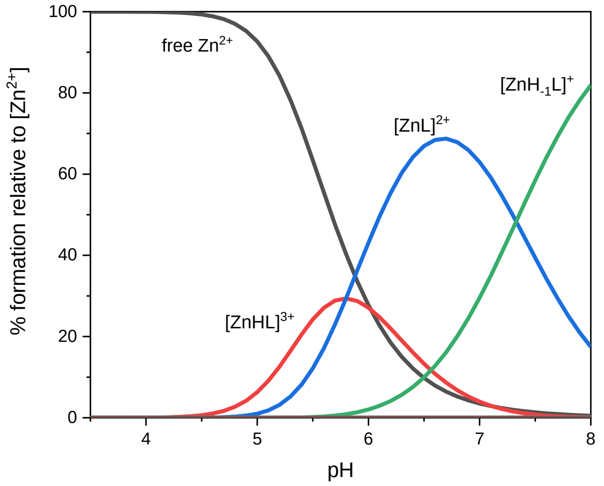

Figure S6. Species distribution diagram of Zn^{2+}/ **L1**; M:L ratio 0.9:1.

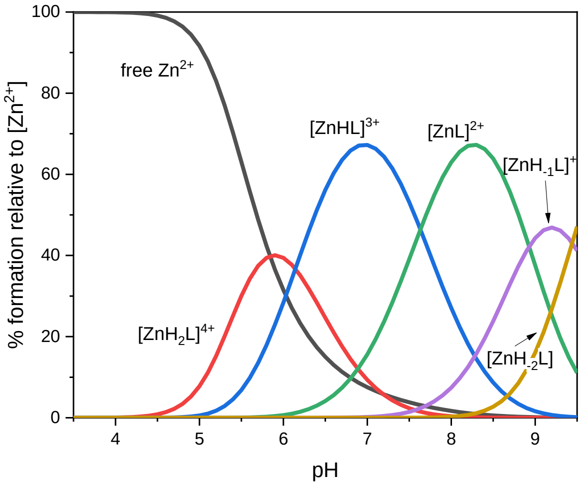

Figure S7. Species distribution diagram of Zn^{2+}/ **L2**; M:L ratio 0.9:1.

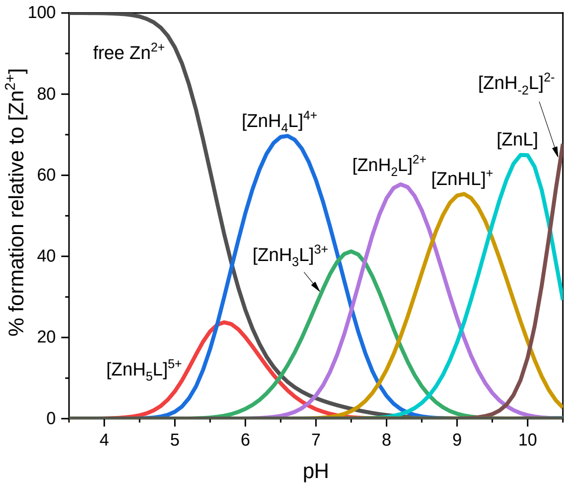

Figure S8. Species distribution diagram of Zn^{2+}/ **L3**; M:L ratio 0.9:1.

4. Spectroscopic data: UV-Vis, CD and EPR

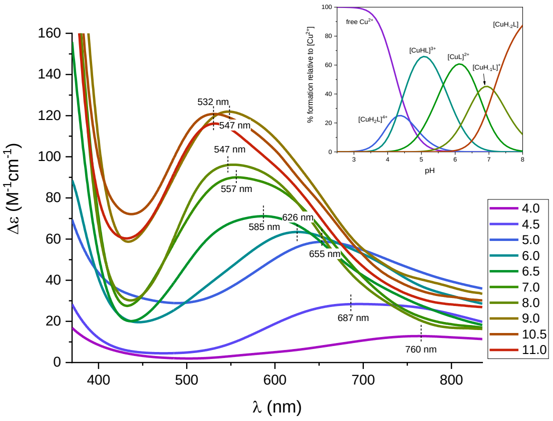

Figure S9. Vis absorption spectra of Cu^{2+} complexes with **L1** at different pH values; M:L ratio 0.9:1. $C_M = 0.45·10^{-3}$ M, optical path 1 cm. The wavelength of maximum absorption is reported for each Vis absorption spectrum.

A) **B)**

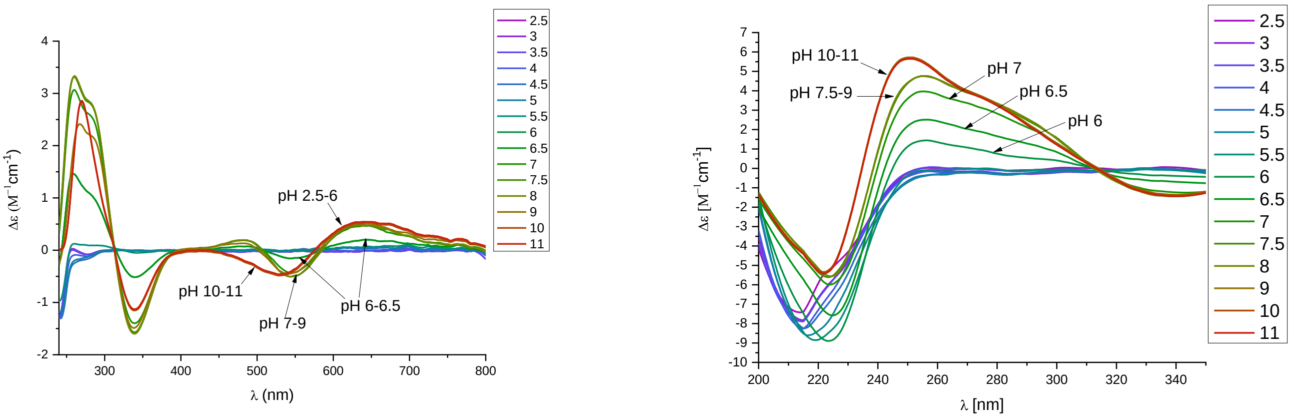

Figure S10. CD spectra of Cu^{2+} complexes with **L1** at different pH values; M:L ratio 0.9:1. $C_M = 0.45·10^{-3}$ M, optical path (A) 1 cm; (B) 0.1 cm.

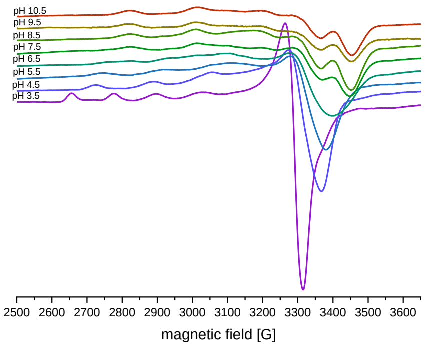

Figure S11. X-band EPR spectra of frozen solution (77 K) of Cu^{2+} complexes with **L1** at different pH values; *I*=0.1 M (KCl), M:L ratio 0.9:1, $C_M = 1.00·10^{-3}$ M.

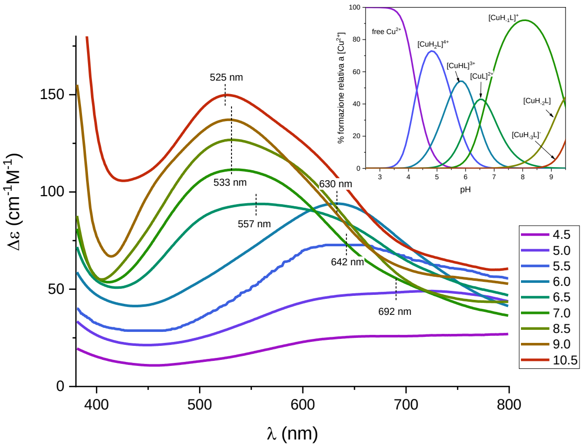

Figure S12. Vis absorption spectra of Cu^{2+} complexes with **L2** at different pH values; M:L ratio 0.9:1. $C_M = 0.45 \cdot 10^{-3}$ M, optical path 1 cm. The wavelength of maximum absorption is reported for each Vis absorption spectrum.

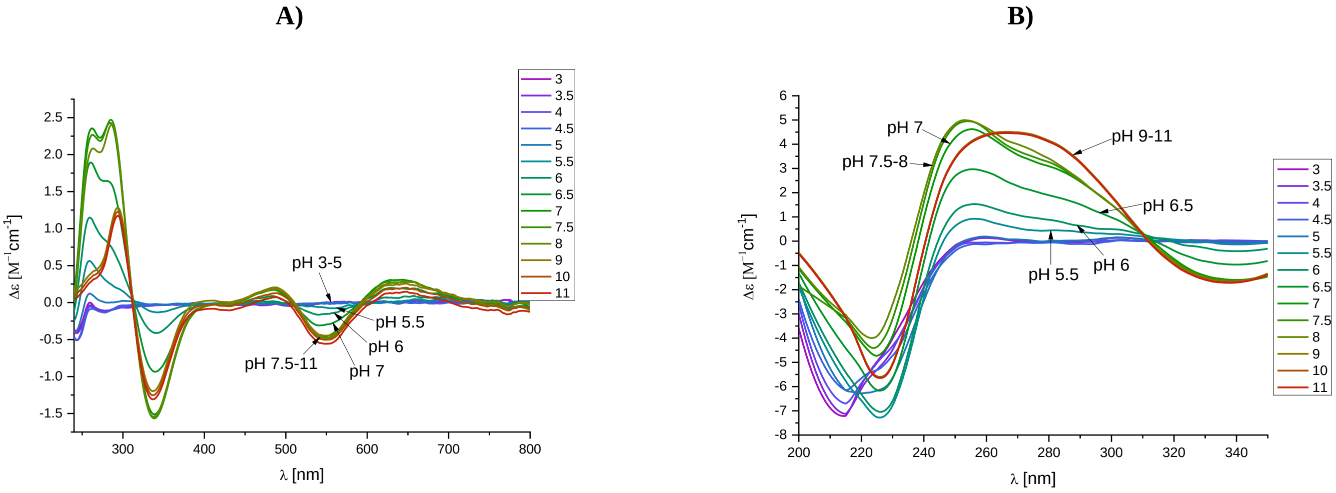

Figure S13. CD spectra of Cu^{2+} complexes with **L2** at different pH values; M:L ratio 0.9:1. $C_M = 0.45 \cdot 10^{-3}$ M, optical path (A) 1 cm; (B) 0.1 cm.

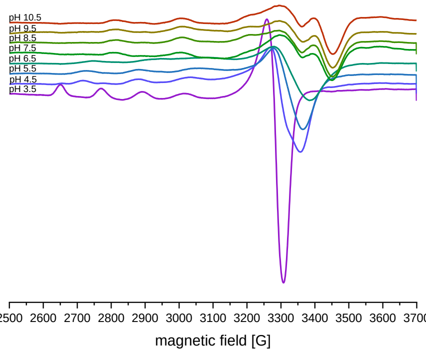

Figure S14. X-band EPR spectra of frozen solution (77 K) of Cu^{2+} complexes with **L2** at different pH values; *I*=0.1 M (KCl), M:L ratio 0.9:1, $C_M = 1.00 \cdot 10^{-3}$ M.

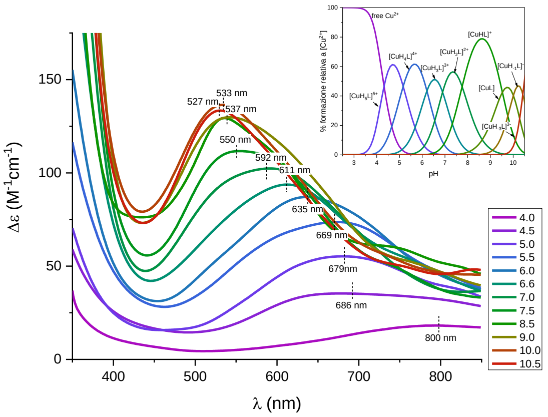

Figure S15. Vis absorption spectra of Cu^{2+} complexes with **L3** at different pH values; M:L ratio 0.9:1. $C_M = 0.45{\cdot}10^{-3}$ M, optical path 1 cm. The wavelength of maximum absorption is reported for each Vis absorption spectrum.

A) **B)**

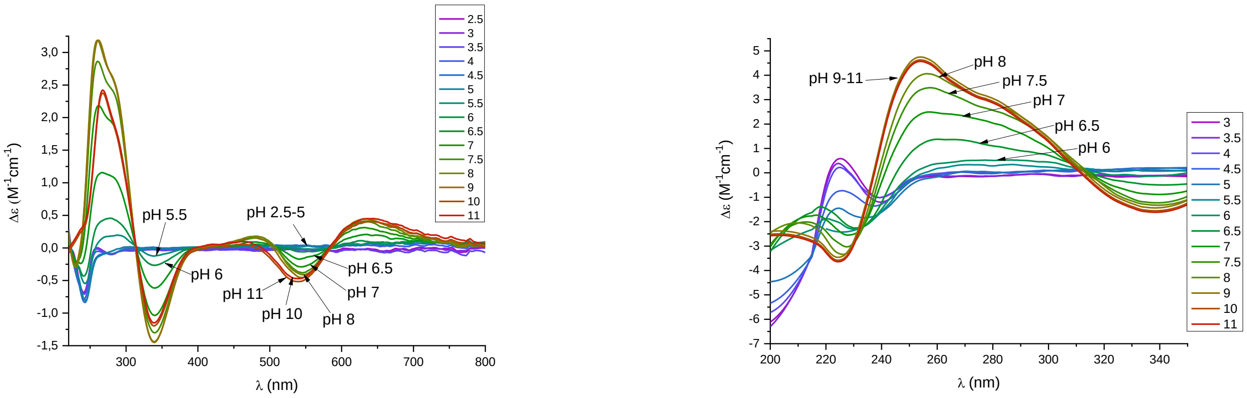

Figure S16. CD spectra of Cu^{2+} complexes with **L3** at different pH values; M:L ratio 0.9:1. $C_M = 0.45{\cdot}10^{-3}$ M, optical path (A) 1 cm; (B) 0.1 cm.

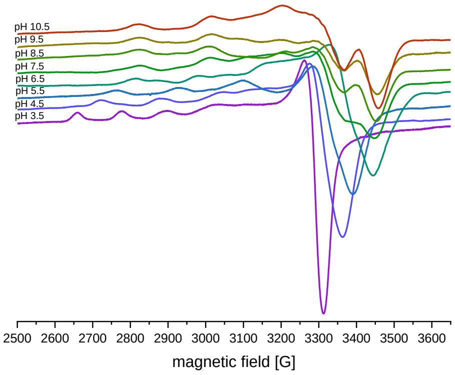

Figure S17. X-band EPR spectra of frozen solution (77 K) of Cu^{2+} complexes with **L3** at different pH values; I=0.1 M (KCl), M:L ratio 0.9:1, $C_M = 1.00{\cdot}10^{-3}$ M.

Table S3. EPR parameters for Cu^{2+} complexes with the studied peptides at I=0.1 M (KCl) and M:L molar ratio = 0.9:1. $C_L = 1.00 \cdot 10^{-3}$ M.

L1: Ac-VAIALKAAHYHTHKE

**pH**	$A_{//}$ [G] (A_z)	$g_{//}$ (g_z)	$g_\perp$ (g_x=g_y)	MW Frequency [GHz]	Coordinated N
**3.5**	121	2.41	2.02	9.58	0
**4.5**	153	2.32	2.02	9.88	1
**5.5**	174	2.28	2.02	9.59	2
**6.5**	176	2.27	2.03	9.58	3
**7.5**	182	2.21	2.03	9.58	4
**8.5**	186	2.21	2.04	9.59	4
**9.5**	190	2.21	2.04	9.59	4
**10.5**	192	2.20	2.05	9.58	4

L2: Ac-VAIALKAAHYHTHKE-NH$_2$

	$A_{//}$ [G] (A_z)	$g_{//}$ (g_z)	$g_\perp$ (g_x=g_y)	MW Frequency [GHz]	Coordinated N
**3.5**	119	2.42	2.02	9.57	0
**4.5**	148	2.32	2.02	9.58	1
**5.5**	157	2.31	2.02	9.57	2
**6.5**	183	2.26	2.02	9.57	3
**7.5**	189	2.21	2.04	9.57	4
**8.5**	190	2.21	2.05	9.58	4
**9.5**	192	2.20	2.05	9.58	4
**10.5**	194	2.20	2.05	9.58	4

L3: VAIALKAAHYHTHKE-NH$_2$

	$A_{//}$ [G] (A_z)	$g_{//}$ (g_z)	$g_\perp$ (g_x=g_y)	MW Frequency [GHz]	Coordinated N
**3.5**	121	2.42	2.02	9.59	0
**4.5**	155	2.32	2.02	9.89	1
**5.5**	163	2.28	2.02	9.58	2
**6.5**	168	2.27	2.02	9.58	2-3
**7.5**	188	2.21	2.02	9.59	3-4
**8.5**	188	2.21	2.03	9.58	4
**9.5**	195	2.20	2.05	9.59	4
**10.5**	198	2.21	2.05	9.59	4

5. Far-UV circular dichroism

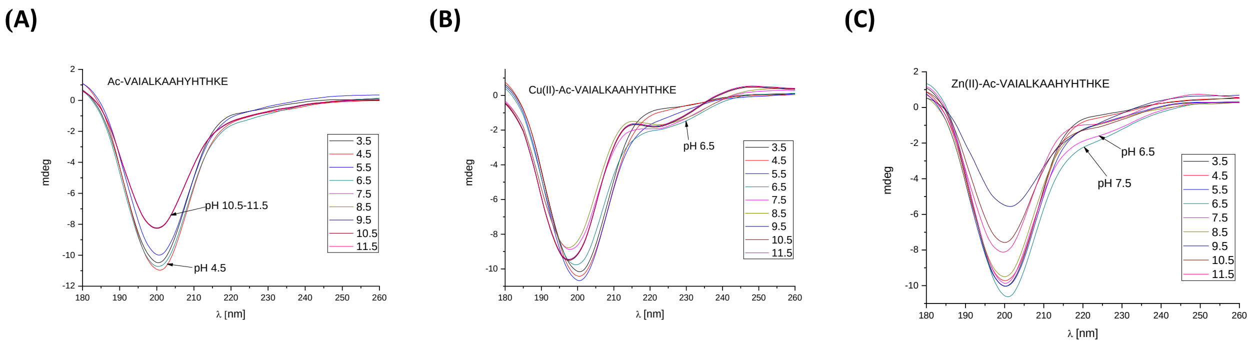

Figure S18. Comparison of CD spectra of (A) **L1** and its (B) Cu^{2+} and (C) Zn^{2+} complexes at T=298 K, I=0.1 M (KCl), M:L ratio 0.9:1, $C_M = 0.45\cdot10^{-3}$ M, optical path 0.01 cm.

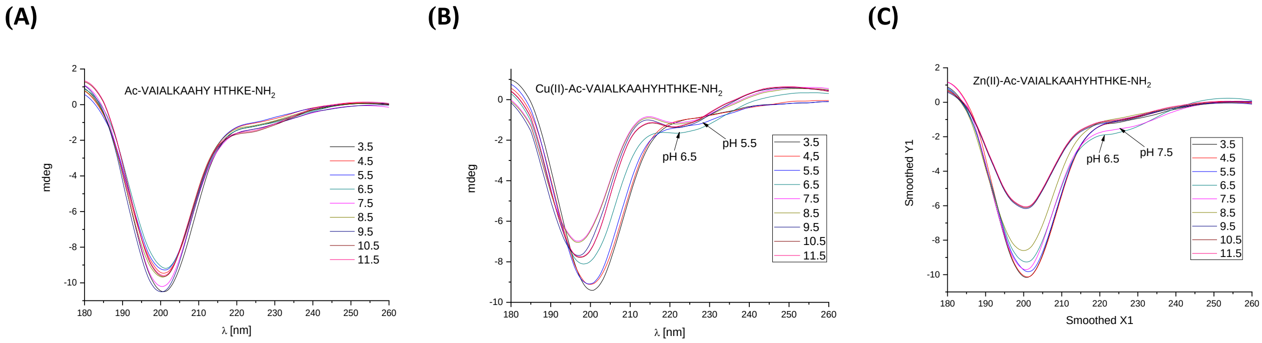

Figure S19. Comparison of CD spectra of (A) **L2** and its (B) Cu^{2+} and (C) Zn^{2+} complexes at T=298 K, I=0.1 M (KCl), M:L ratio 0.9:1, $C_M = 0.45\cdot10^{-3}$ M, optical path 0.01 cm.

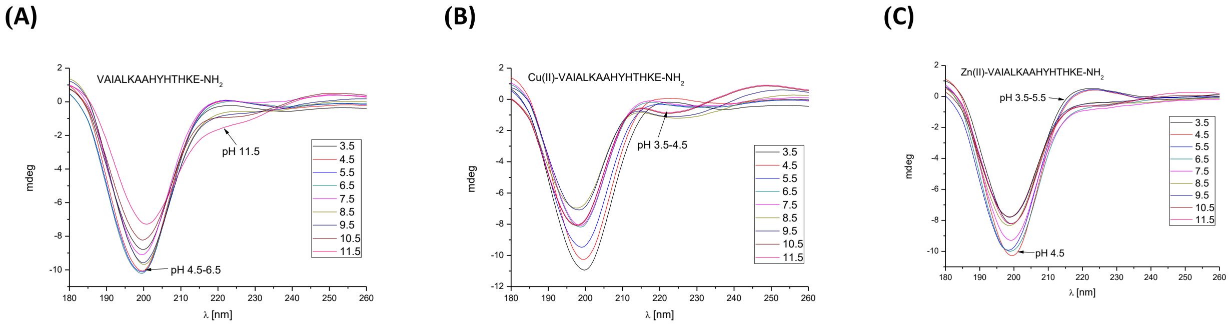

Figure S20. Comparison of CD spectra of (A) **L3** and its (B) Cu^{2+} and (C) Zn^{2+} complexes at T=298 K, I=0.1 M (KCl), M:L ratio 0.9:1, $C_M = 0.45\cdot10^{-3}$ M, optical path 0.01 cm.

6. Mass spectrometry

Table S4. Stoichiometry, molecular formula and average *m/z* value for the species present in ESI-MS spectra of Cu^{2+} and Zn^{2+} complexes with the studied ligands; L:M molar ratio = 1:1 in MeOH:H_2O (1:1) mixture solution.

	**pH**	**Species**	**Formula**	**Average *m/z***
**L1** Positive mode	5.2	H_3L^{3+}	C79H124N23O20	577.9
		$[CuHL]^{3+}$	C79H122N23O20Cu	598.6
		$([CuL] \cdot K)^{3+}$	C79H121N23O20CuK	611.3
		$[ZnHL]^{3+}$	C79H122N23O20Zn	599.3
	7.4	H_3L^{3+}	C79H124N23O20	577.6
		$[CuHL]^{3+}$	C79H122N23O20Cu	598.6
		$([CuL] \cdot K)^{3+}$	C79H121N23O20CuK	611.3
		$[ZnHL]^{3+}$	C79H122N23O20Zn	599.3
**L2** Positive mode	5.2	H_3L^{3+}	C79H123N24O19	577.7
		H_2L^{2+}	C79H122N24O19	865.5
		$(HL \cdot Na)^{2+}$	C79H121N24O19Na	877.0
		$(HL \cdot K)^{2+}$	C79H121N24O19K	885.4
		$[CuHL]^{3+}$	C79H121N24O19Cu	598.3
		$[ZnL]^{2+}$	C79H120N24O19Zn	898.4
	7.4	H_3L^{3+}	C79H123N24O19	577.7
		H_2L^{2+}	C79H122N24O19	865.5
		$(HL \cdot Na)^{2+}$	C79H121N24O19Na	877.0
		$(HL \cdot K)^{2+}$	C79H121N24O19K	885.4
		$[CuHL]^{3+}$	C79H121N24O19Cu	598.3
		$([CuL] \cdot K)^{3+}$	C79H120N24O19CuK	611.3
		$[ZnL]^{2+}$	C79H120N24O19Zn	898.4
**L3** Positive mode	5.2	H_5L^{3+}	C77H125N24O19	563.3
		$[CuH_3L]^{3+}$	C77H123N24O19Cu	584.3
		$([CuH_2L] \cdot K)^{3+}$	C77H122N24O19CuK	597.1
		$[ZnH_3L]^{3+}$	C77H123N24O19Zn	584.6
		$([ZnH_2L] \cdot K)^{3+}$	C77H122N24O19ZnK	597.3
	7.4	H_5L^{3+}	C77H125N24O19	563.3
		$(H_4L \cdot Na)^{3+}$	C77H124N24O19Na	571.0
		$[CuH_3L]^{3+}$	C77H123N24O19Cu	584.3
		$([CuH_2L] \cdot K)^{3+}$	C77H122N24O19CuK	597.1
		$[ZnH_3L]^{3+}$	C77H123N24O19Zn	584.6

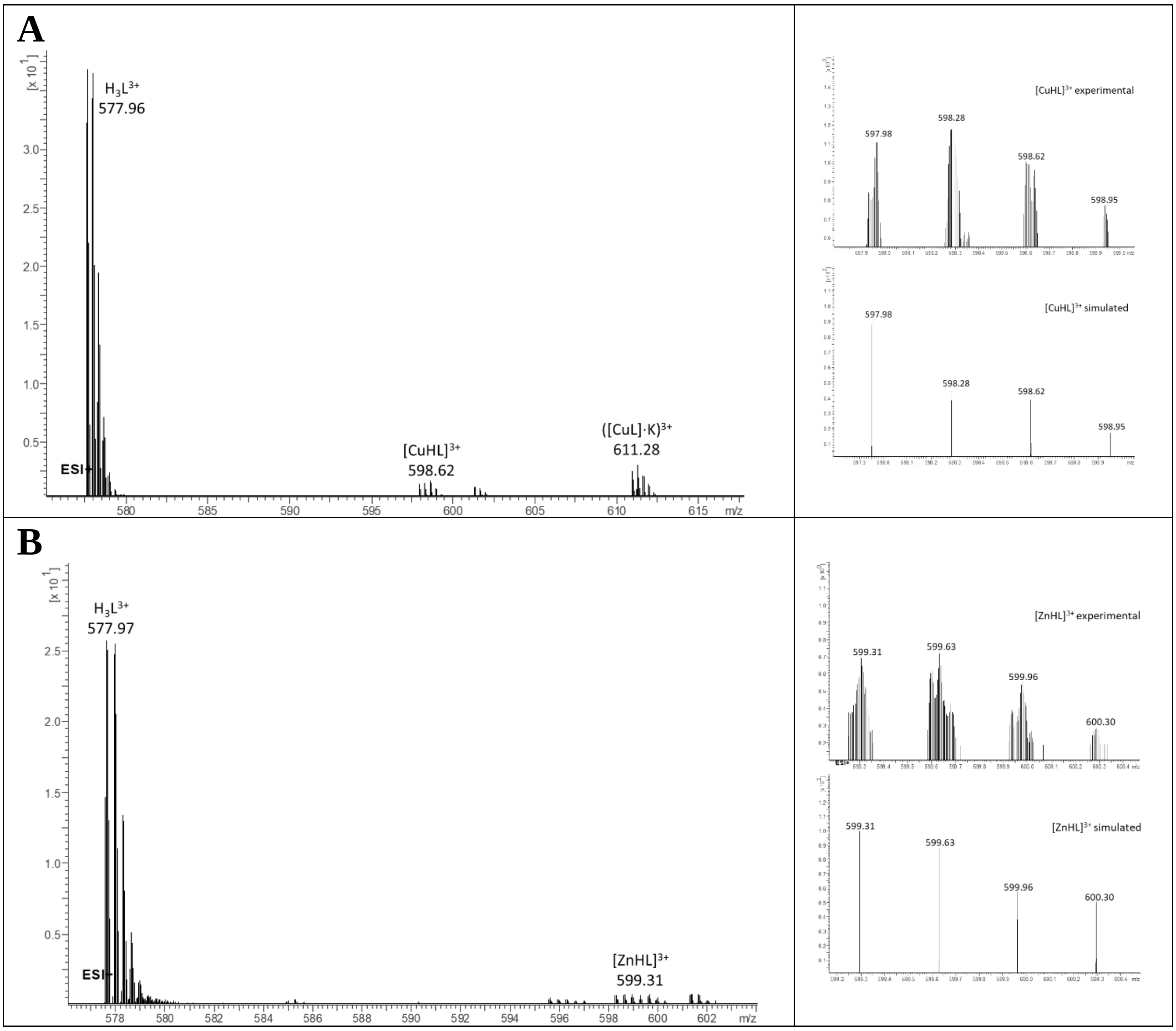

Figure S21. (A) *Left*: ESI-MS spectrum for Cu^{2+}/**L1** system at L:M molar ratio=1:1 in MeOH:H_2O (1:1) mixture solution, pH 5.2. *Right*: Comparison of experimental and simulated isotopic pattern of the chosen metal complex $[CuH_3L]^{3+}$. (B) *Left*: ESI-MS spectrum for Zn^{2+}/**L1** system at L:M molar ratio=1:1 in MeOH:H_2O (1:1) mixture solution, pH 7.4. *Right*: Comparison of experimental and simulated isotopic pattern of the chosen metal complex $[ZnH_3L]^{3+}$.

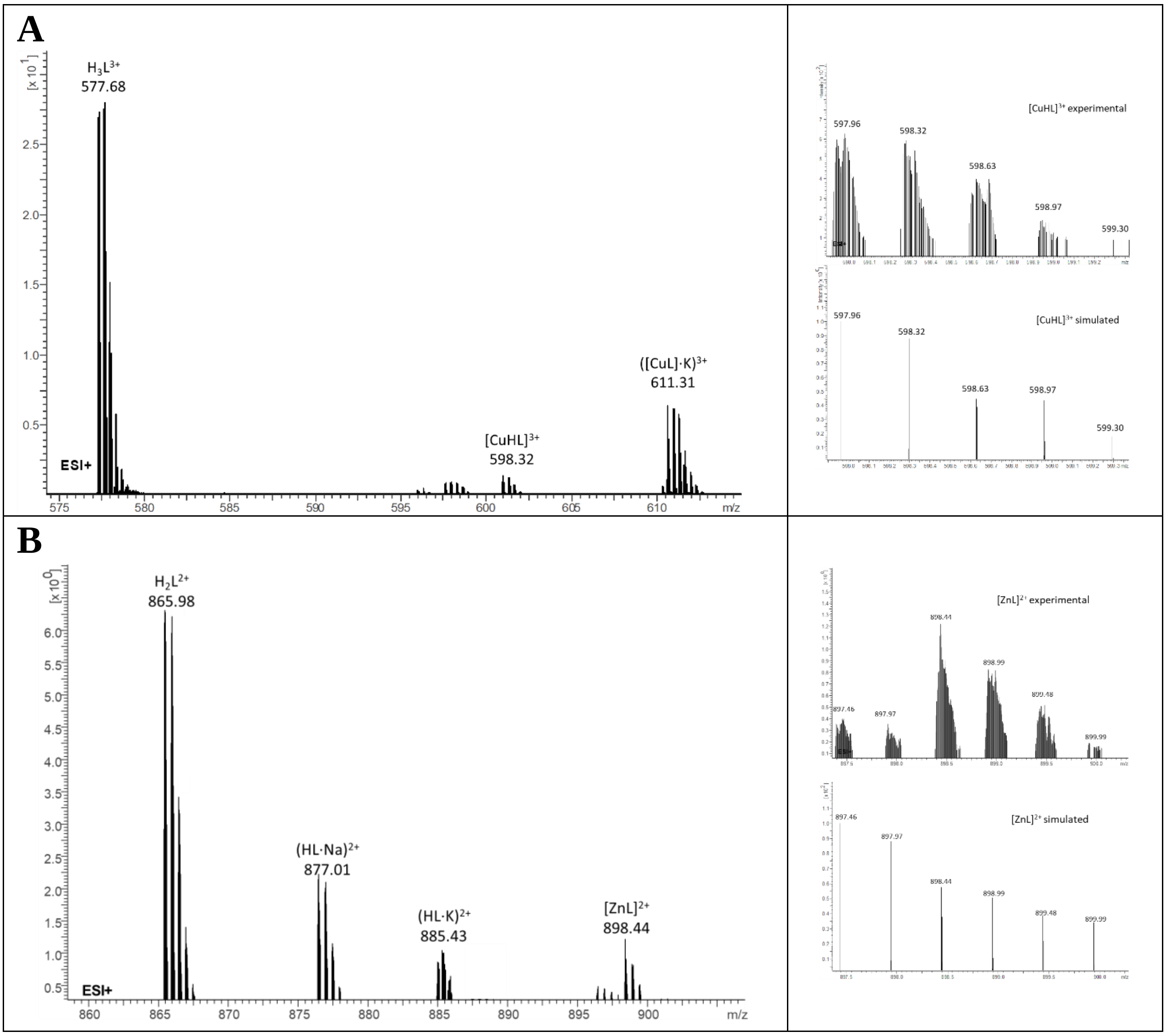

Figure S22. (A) *Left*: ESI-MS spectrum for Cu^{2+}/**L2** system at L:M molar ratio=1:1 in MeOH:H_2O (1:1) mixture solution, pH 7.4. *Right*: Comparison of experimental and simulated isotopic pattern of the chosen metal complex $[CuHL]^{3+}$. (B) *Left*: ESI-MS spectrum for Zn^{2+}/**L2** system at L:M molar ratio=1:1 in MeOH:H_2O (1:1) mixture solution, pH 7.4. *Right*: Comparison of experimental and simulated isotopic pattern of the chosen metal complex $[ZnL]^{2+}$.

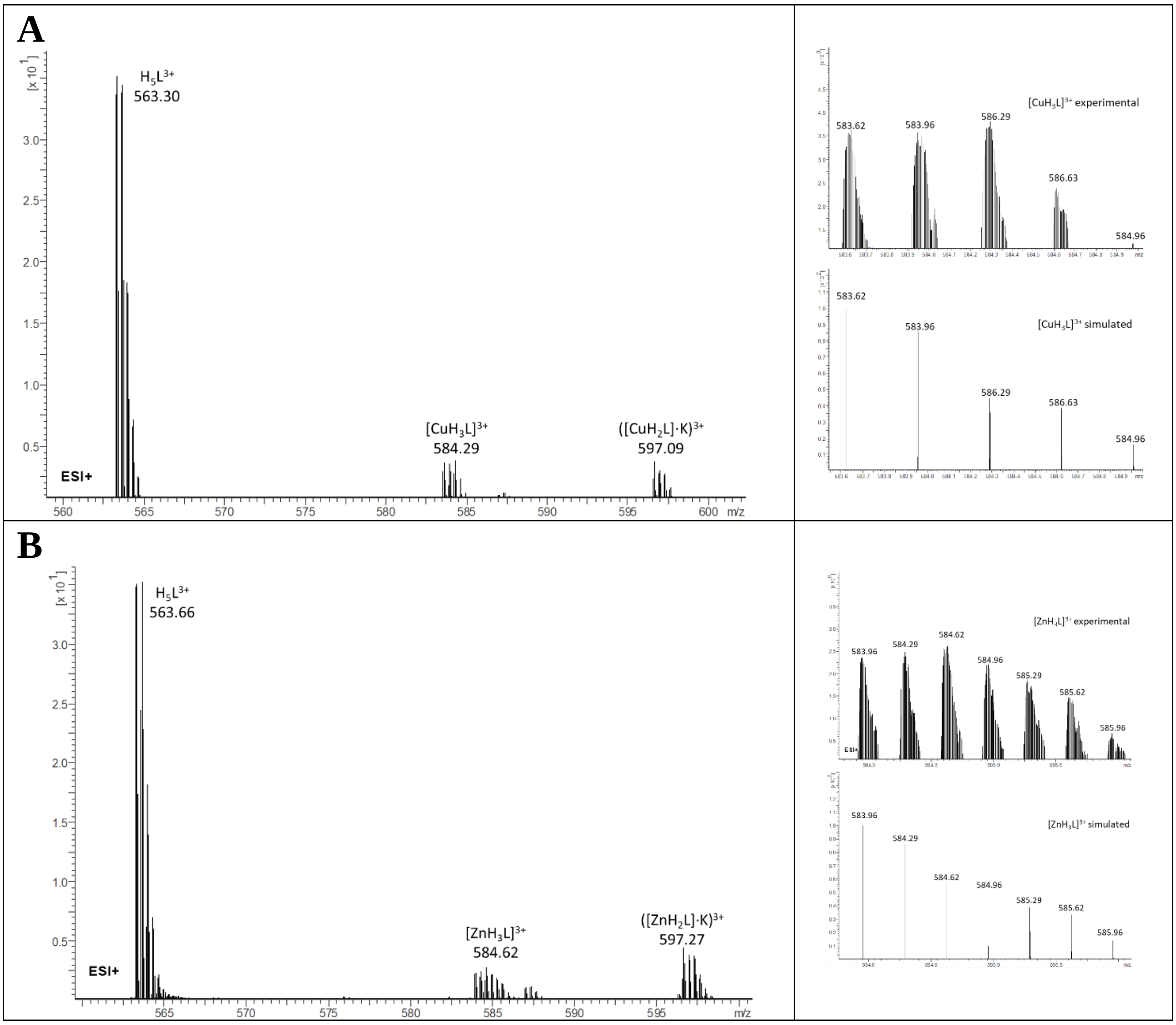

Figure S23. (A) *Left*: ESI-MS spectrum for Cu^{2+}/**L3** system at L:M molar ratio=1:1 in MeOH:H_2O (1:1) mixture solution, pH 7.4. *Right*: Comparison of experimental and simulated isotopic pattern of the chosen metal complex $[CuHL]^{3+}$. (B) *Left*: ESI-MS spectrum for Zn^{2+}/**L3** system at L:M molar ratio=1:1 in MeOH:H_2O (1:1) mixture solution, pH 5.2. *Right*: Comparison of experimental and simulated isotopic pattern of the chosen metal complex $[ZnL]^{2+}$.

7. Competition plots

These graphs are based on the experimental stability constants reported above and represent a simulation of solutions containing equimolar concentrations of the metal and two or more ligands, assuming that all peptides compete to bind the metal and form only the binary complexes described in the speciation model.

A

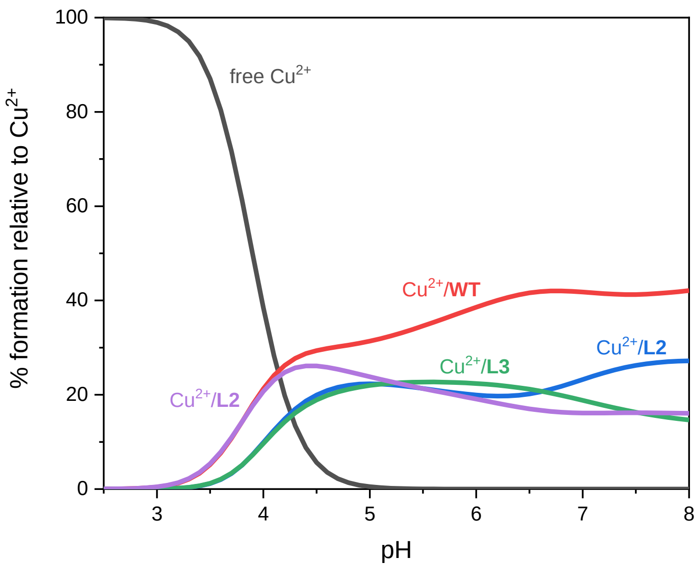

B

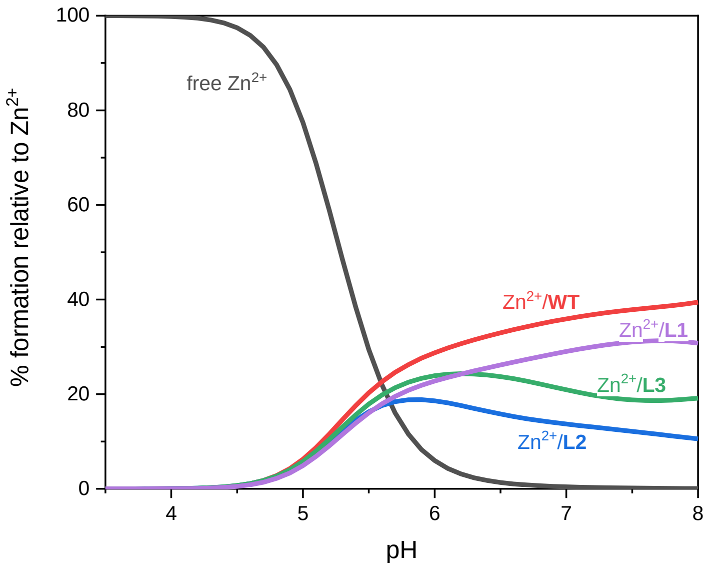

Figure S24. Competition plots for a simulated solution containing equimolar concentrations of (A) Cu^{2+}, (B) Zn^{2+}, and **WT, L1, L2** and **L3**.

8. Antimicrobial activity

Table S5. Effect of **WT** calcitermin and **L1**, **L2**, **L3** derivatives on *Candida albicans* (ATCC 10231) proliferation.

		**Incubation time**					
		**3 hours**			**24 hours**		
	**Condition**	**Peptide [c]**			**Peptide [c]**		
		**0.032 mg/mL**	**0.064 mg/mL**	**0.128 mg/mL**	**0.032 mg/mL**	**0.064 mg/mL**	**0.128 mg/mL**
**WT calcitermin**	**None (CTR)**	84.3 ± 22	84.3 ± 22	84.3 ± 22	1,779.8 ± 95	1,779.8 ± 95	1,779.8 ± 95
	**Peptide**	28 ± 15 (-67%)	28 ± 15 (-67%)	22.4 ± 15 (-73%)	1,348± 698 (-24%)	1,289 ± 495 (-33%)	1,052 ± 531 (-41%)
	**Peptide + $ZnCl_2$**	67 ± 35 (-20%)	48.3 ± 22 (-43%)	42.7 ± 11 (-49%)	1,235.8 ± 235 (-31%)	754.3 ± 323 (-58%)	62.8 ± 33 (-96%)
	**Peptide + $CuCl_2$**	74 ± 28 (-12%)	85 ± 24 (+1%)	55 ± 11 (-35%)	1,771 ± 309 (-0.4%)	1,489 ± 111 (-16%)	1,790 ± 393 (-0.6%)
	**$ZnCl_2$**	94.2 ± 23 (+12%)	75.33 ± 25 (-11%)	34.7 ± 9 (-59%)	1,413.7 ± 348 (-21%)	1,143± 571 (-36%)	116.2 ± 85 (-93%)
	**$CuCl_2$**	90 ± 3 (+6.8%)	88 ± 5 (+4.4%)	84 ± 3 (0%)	1,800 ± 195 (+1.1%)	1,820 ± 125 (+2.2%)	1,816 ± 109 (+2%)
**L1**	**None (CTR)**	55 ± 7	55 ± 7	55 ± 7	2,177 ± 225	2,177 ± 225	2,177 ± 225
	**Peptide**	40 ± 10 (- 27%)	54 ± 6 (-1.8%)	27 ± 0.6 (-51%)	1,789 ± 225 (-18%)	1,807 ± 433 (-17%)	1,666 ± 229 (-23%)
	**Peptide + $ZnCl_2$**	67 ± 4 (+16%)	88 ± 7 (+60%)	72 ± 10 (+31%)	2,230 ± 326 (-2%)	1,619 ± 165 (-26%)	265 ± 108 (-88%)
	**Peptide + $CuCl_2$**	40 ± 28 (-27.3%)	40 ± 24 (-27.3%)	40 ± 11 (-27.3%)	1,908 ± 160 (-12%)	1,576 ± 112 (-28%)	1,428 ± 24 (-34%)
	**$ZnCl_2$**	56 ± 6 (+12%)	49 ± 2 (-11%)	32 ± 9 (-42%)	1,915 ± 234 (-12%)	1,307 ± 360 (-40%)	346 ± 76 (-84%)
	**$CuCl_2$**	60 ± 3 (+9%)	58 ± 5 (+5.4%)	49 ± 3 (-11%)	2,184 ± 195 (+0.3%)	2,152 ± 228 (-1.1%)	2,139 ± 55 (-2%)
**L2**	**None (CTR)**	140 ± 16	140 ± 16	140 ± 16	1,639 ± 72	1,639 ± 72	1,639 ± 72
	**Peptide**	211 ± 25 (+51%)	168 ± 14 (+20%)	148 ± 24 (+5.7%)	1,563 ± 72 (-5%)	1,480 ± 198 (-10%)	1,732 ± 249 (+6%)
	**Peptide + $ZnCl_2$**	129 ± 17 (-7.8%)	79 ± 18 (-44%)	36 ± 15 (-74%)	1,873 ± 83 (+14%)	881 ± 99 (-46%)	50 ± 29 (-97%)
	**Peptide + $CuCl_2$**	153 ± 26 (+9.3%)	180 ± 81 (+29%)	130 ± 29 (-7%)	1,908 ± 83 (+16%)	1,360 ± 140 (-17%)	1,256 ± 129 (-23%)
	**$ZnCl_2$**	91 ± 25 (-35%)	87 ± 6 (-38%)	62 ± 6 (-56%)	1,797 ± 162 (+10%)	984 ± 28 (-40%)	93 ± 61 (-94%)
	**$CuCl_2$**	153 ± 26 (+9%)	160 ± 81 (+14%)	135 ± 29 (-3.4%)	1,671 ± 25 (+1.9%)	1,697 ± 81 (+3%)	1,360 ± 68 (-17%)
**L3**	**None (CTR)**	151 ± 11	151 ± 11	151 ± 11	2,515 ± 533	2,515 ± 533	2,515 ± 533
	**Peptide**	189 ± 23 (+25%)	182 ± 27 (+20%)	144 ± 11 (-5%)	2,263 ± 251 (-10%)	2,204 ± 170 (-12%)	2,164 ± 101 (-14%)
	**Peptide + $ZnCl_2$**	160 ± 25 (+6%)	148 ± 22 (-2%)	71 ± 17 (-51%)	2,557 ± 251 (+2%)	1,596 ± 145 (-37%)	605 ± 97 (-76%)
	**Peptide + $CuCl_2$**	187 ± 20 (+23%)	241 ± 23 (+59%)	230 ± 3 (+53%)	2,042 ± 137 (-19%)	2,315 ± 546 (-8%)	1,687 ± 111 (-33%)
	**$ZnCl_2$**	107 ± 13 (-29%)	98 ± 15 (-35%)	53 ± 14 (-64.9%)	2,249 ± 145 (-11%)	1563 ± 29 (-38%)	573 ± 87 (-77%)
	**$CuCl_2$**	180 ± 15 (+19%)	174 ± 22 (+15%)	159 ± 31 (+5.3%)	2,558 ± 367 (+1.7%)	2,456 ± 236 (-2.3%)	1,946 ± 96 (-23%)

The results are expressed as mean CFU number ± S.D. corresponding to 0.1 mL of microbial suspension after 24 hours of incubation at 37°C, and derive from triplicate samples in two independent experiments. Percentages of decrease/increase with respect to untreated controls are also reported in parentheses. $ZnCl_2$ and $CuCl_2$ were added at a molar ratio 0.9:1 with respect to peptides. The same $ZnCl_2$ and $CuCl_2$ concentrations were used without peptides, as controls. CTR, untreated control.

Table S6. Effect of **WT** Calcitermin and **L1**, **L2**, **L3** derivatives on *Escherichia coli* (ATCC 25922) proliferation.

	Condition	Incubation time					
		3 hours			24 hours		
		Peptide [c]			Peptide [c]		
		0.032 mg/mL	0.064 mg/mL	0.128 mg/mL	0.032 mg/mL	0.064 mg/mL	0.128 mg/mL
**WT calcitermin**	**None (CTR)**	812 ± 117	812 ± 117	812 ± 117	241 ± 11	241 ± 11	241 ± 11
	**Peptide**	718 ± 138 (-12%)	659 ± 139 (-19%)	1155 ± 270 (+42%)	224 ± 21 (-7%)	275 ± 16 (+14%)	233 ± 10 (-3%)
	**Peptide + $ZnCl_2$**	499 ± 104 (-38%)	368 ± 18 (-55%)	74 ± 17 (-91%)	224 ± 8 (-7%)	249 ± 9 (+3%)	222 ± 16 (-8%)
	**Peptide + $CuCl_2$**	505 ± 26 (-38%)	360 ± 97 (-56%)	72 ± 44 (-91.1%)	191 ± 23 (-21%)	215 ± 17 (-11%)	42 ± 12 (-82%)
	**$ZnCl_2$**	449 ± 74 (-45%)	329 ± 11 (-59%)	72 ± 21 (-91%)	238 ± 16 (-1%)	235 ± 6 (-3%)	168 ± 22 (-30%)
	**$CuCl_2$**	474 ± 91 (-42%)	383 ± 82 (-53%)	64 ± 24 (-92%)	256 ± 3 (+6%)	47 ± 28 (-80%)	9 ± 5 (-96%)
**L1**	**None (CTR)**	1,047 ± 172	1,047 ± 172	1,047 ± 172	172 ± 10	172 ± 10	172 ± 10
	**Peptide**	749 ± 47 (-28%)	634 ± 10 (-39%)	625 ± 76 (-40%)	174 ± 6 (+1%)	146 ± 17 (-15%)	140 ± 11 (-19%)
	**Peptide + $ZnCl_2$**	343 ± 13 (-67%)	198 ± 7 (-81%)	45 ± 18 (-96%)	163 ± 18 (-6%)	149 ± 7 (-13%)	187 ± 12 (+8%)
	**Peptide + $CuCl_2$**	372 ± 16 (-65%)	385 ± 24 (-63%)	151 ± 30 (-85%)	147 ± 6 (-15%)	58 ± 8 (-67%)	17 ± 18 (-90%)
	**$ZnCl_2$**	559 ± 25 (-47%)	249 ± 11 (-76%)	49 ± 8 (-95%)	170 ± 14 (-1%)	163 ± 47 (-5%)	168 ± 22 (-3%)
	**$CuCl_2$**	665 ± 96 (-36%)	255 ± 23 (-76%)	57 ± 4 (-95%)	160 ± 42 (-7%)	93 ± 28 (-46%)	42 ± 16 (-76%)
**L2**	**None (CTR)**	1,047 ± 172	1,047 ± 172	1,047 ± 172	172 ± 10	172 ± 10	172 ± 10
	**Peptide**	1,037 ± 161 (-1%)	1031 ± 83 (-2%)	845 ± 55 (-19%)	187 ± 11 (+9%)	186 ± 14 (+8%)	166 ± 20 (-3%)
	**Peptide + $ZnCl_2$**	378 ± 8 (-64%)	248 ± 88 (-76%)	44 ± 4 (-96%)	150 ± 4 (-12.7%)	146 ± 6 (-15.1%)	158 ± 18 (-8.1%)
	**Peptide + $CuCl_2$**	467 ± 19 (-55%)	371 ± 54 (-65%)	190 ± 15 (-82%)	167 ± 37 (-2.9%)	167 ± 12 (-2.9%)	136 ± 25 (-21%)
	**$ZnCl_2$**	559 ± 25 (-47%)	249 ± 11 (-76%)	49 ± 8 (-95%)	170 ± 14 (-1%)	163 ± 47 (-5%)	168 ± 22 (-3%)
	**$CuCl_2$**	665 ± 96 (-36%)	255 ± 23 (-76%)	57 ± 4 (-95%)	160 ± 42 (-7%)	93 ± 28 (-46%)	42 ± 16 (-76%)
**L3**	**None (CTR)**	1,047 ± 172	1,047 ± 172	1,047 ± 172	172 ± 10	172 ± 10	172 ± 10
	**Peptide**	810 ± 100 (-23%)	737 ± 147 (-30%)	893 ± 113 (-15%)	202 ± 18 (+17%)	166 ± 8 (-3%)	193 ± 9 (+12%)
	**Peptide + $ZnCl_2$**	439 ± 91 (-58%)	224 ± 4 (-79%)	59 ± 9 (-94%)	202 ± 23 (+17%)	186 ± 21 (+8%)	187 ± 17 (+9%)
	**Peptide + $CuCl_2$**	509 ± 100 (-51%)	198 ± 117 (-81%)	191 ± 7 (-82%)	184 ± 17 (+7%)	138 ± 48 (-20%)	102 ± 20 (-41%)
	**$ZnCl_2$**	559 ± 25 (-47%)	249 ± 11 (-76%)	49 ± 8 (-95%)	170 ± 14 (-1%)	163 ± 47 (-5%)	168 ± 22 (-3%)
	**$CuCl_2$**	665 ± 96 (-36%)	255 ± 23 (-76%)	57 ± 4 (-95%)	160 ± 42 (-7%)	93 ± 28 (-46%)	42 ± 16 (-76%)

The results are expressed as mean CFU number ± S.D. corresponding to 0.1 mL of microbial suspension after 24 hours of incubation at 37°C, and derive from triplicate samples in two independent experiments. Percentages of decrease/increase with respect to untreated controls are also reported in parentheses. $ZnCl_2$ and $CuCl_2$ were added at a molar ratio 0.9:1 with respect to peptides. The same $ZnCl_2$ and $CuCl_2$ concentrations were used without peptides, as controls. CTR, untreated control.

Table S7. Effect of **WT** Calcitermin and **L1**, **L2**, **L3** derivatives on *Staphylococcus aureus* (ATCC 25923) proliferation.

	Condition	Incubation time 3 hours Peptide [c] 0.032 mg/mL	0.064 mg/mL	0.128 mg/mL
**WT calcitermin**	**None (CTR)**	161 ± 27	161 ± 27	161 ± 27
	**Peptide**	146 ± 7 (-9.3%)	159 ± 45 (-1.2%)	158 ± 59 (-1.8%)
	**Peptide + $ZnCl_2$**	169 ± 40 (+5%)	139 ± 20 (-14%)	96 ± 21 (-41%)
	**Peptide + $CuCl_2$**	209 ± 44 (+30%)	175 ± 30 (+9%)	229 ± 55 (+42%)
	**$ZnCl_2$**	178 ± 60 (+10%)	100 ± 11 (-37.8%)	86 ± 40 (-47%)
	**$CuCl_2$**	201 ± 25 (+25%)	188 ± 62 (+16%)	201 ± 23 (+25%)
**L1**	**None (CTR)**	161 ± 27	161 ± 27	161 ± 27
	**Peptide**	215 ± 68 (+34%)	155 ± 31 (-4%)	211 ± 49 (+31%)
	**Peptide + $ZnCl_2$**	183 ± 24 (+14%)	191 ± 6 (+19%)	87 ± 8 (-46%)
	**Peptide + $CuCl_2$**	193 ± 54 (+20%)	221 ± 66 (+37%)	166 ± 35 (+3%)
	**$ZnCl_2$**	125 ± 60 (-22.4%)	100 ± 11 (-37.8%)	86 ± 40 (-47%)
	**$CuCl_2$**	201 ± 25 (+25%)	188 ± 62 (+16%)	201 ± 23 (+25%)
**L2**	**None (CTR)**	177 ± 18	177 ± 18	177 ± 18
	**Peptide**	157 ± 35 (-12%)	201 ± 40 (+14%)	213 ± 23 (+21%)
	**Peptide + $ZnCl_2$**	177 ± 11 (0%)	147 ± 16 (-17%)	82 ± 16 (-54%)
	**Peptide + $CuCl_2$**	129± 41 (-17%)	127 ± 30 (-18%)	194 ± 4 (+6%)
	**$ZnCl_2$**	121 ± 11 (-31%)	84 ± 13 (-53%)	41 ± 5 (-77%)
	**$CuCl_2$**	178 ± 2 (+0.5%)	170 ±17 (-4%)	159 ± 22 (-10%)
**L3**	**None (CTR)**	177 ± 18	177 ± 18	177 ± 18
	**Peptide**	127 ± 64 (-28%)	101 ± 30 (-43%)	91 ± 12 (-49%)
	**Peptide + $ZnCl_2$**	167 ± 22 (-6%)	88 ± 18 (-50%)	48 ± 8 (-73%)
	**Peptide + $CuCl_2$**	187 ± 13 (+6%)	173 ± 69 (-2%)	94 ± 2 (-47%)
	**$ZnCl_2$**	121 ± 11 (-31%)	84 ± 13 (-53%)	41 ± 5 (-77%)
	**$CuCl_2$**	178 ± 2 (+0.5%)	170 ±17 (-4%)	159 ± 22 (-10%)

The results are expressed as mean CFU number ± S.D. corresponding to 0.1 mL of microbial suspension after 24 hours of incubation at 37°C, and derive from triplicate samples in two independent experiments. Percentages of decrease/increase with respect to untreated controls are also reported in parentheses. $ZnCl_2$ and $CuCl_2$ were added at a molar ratio 0.9:1 with respect to peptides. The same $ZnCl_2$ and $CuCl_2$ concentrations were used without peptides, as controls. CTR, untreated control.

9. Peptide stability in human plasma

Stability in human plasma has been also tested for the metal complexed form of the *N*-terminal protected analogue of calcitermin (**L1**). The degradation profile of **WT**-calcitermin is reported as a reference.

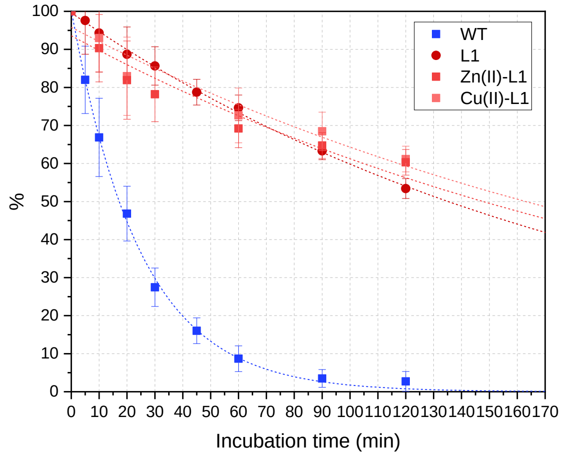

Figure S25. Stability in human plasma of wild-type calcitermin **WT**, its *N*-terminal protected analogue **L1** and the Zn^{2+} and Cu^{2+} complexes with **L1**.

10. CD assay for albumin-peptide systems

The interaction between bovine serum albumin (BSA) and peptides has been qualitatively evaluated by means of circular dichroism experiments in phosphate buffer 0.05 M (pH 7.4). Samples of BSA, and BSA+peptide/complex were prepared by mixing equimolar amounts of BSA and each peptide or copper complex (metal:peptide ratio 1:1) to a final concentration of $C_{BSA}=C_{peptide/complex}=7.5\cdot10^{-5}$ M. The CD spectrum of BSA consists of two negative bands at 209 and 222 nm, characteristic of a α-helical structure. A change in the helical content of BSA is observed in all the systems when the peptide or the copper complex is added to the solution. On the other hand, there are no significant differences between calcitermin and its **L1** derivative in terms of interaction with BSA, nor for the corresponding copper complexes. This result suggests that the interaction with albumin has only a negligible influence on the stability difference between calcitermin and **L1** in human plasma."

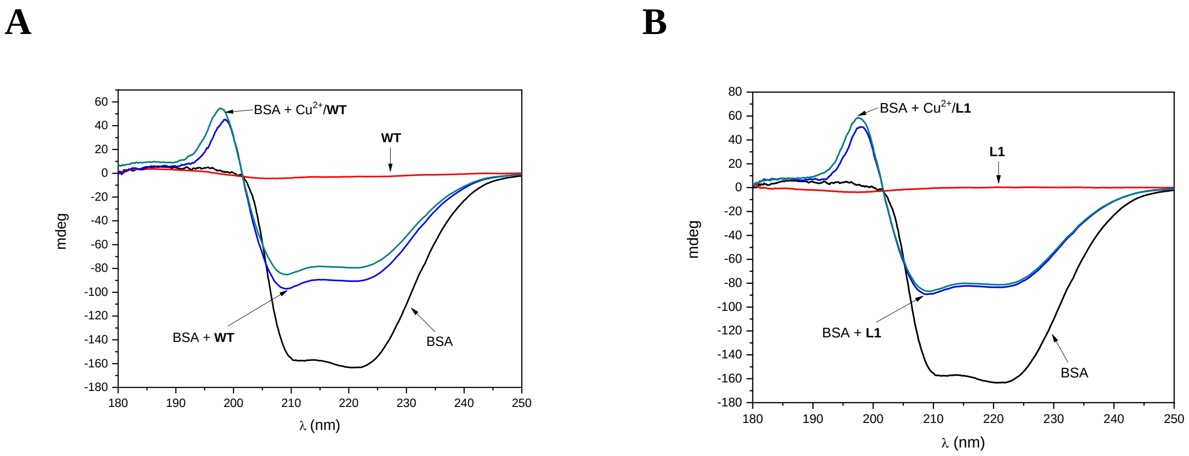

Figure S26. Comparison of CD spectra of BSA in presence and absence of (A) **WT** and (B) **L1**, and their Cu^{2+} complexes at *T*=298 K, phosphate buffer (pH 7.4), BSA:peptide:metal ratio 1:1:1, $C_{BSA}= 7.5\cdot10^{-5}$ M, optical path 0.01 cm.

11. Materials

Employed solutions

$ZnCl_2$ and $CuCl_2$ were extra pure products (Sigma-Aldrich); the concentrations of their stock solutions were standardised by EDTA titration and periodically checked via ICP-MS. The carbonate-free stock solutions of 0.1 M KOH were prepared by diluting concentrated KOH (Sigma-Aldrich) and then potentiometrically standardized with potassium hydrogen phthalate (99.9% purity), as primary standard. All sample solutions were prepared with fresh Milli-Q® water. The HCl and HNO_3 stock solutions were prepared by diluting concentrated HCl and HNO_3 (Sigma-Aldrich) and then standardized with standard KOH. The ionic strength was adjusted to 0.1 M by adding KCl (Sigma-Aldrich). Sample solutions of metal-peptide complexes were prepared with metal:ligand ratio 0.9:1. Grade A glassware was always employed. The bovine serum albumin (BSA) used for CD assay was purchased from Sigma-Aldrich.

Peptide synthesis and purification

Wild-type calcitermin (VAIALKAAHYHTHKE, **WT**) and its N- and C- terminally protected derivatives (Ac-VAIALKAAHYHTHKE, **L1**; Ac-VAIALKAAHYHTHKE-NH_2, **L2**; VAIALKAAHYHTHKE-NH_2, **L3**) were synthesized according to published methods[4] using Fmoc/t-butyl chemistry with a Syro XP multiple peptide synthesizer (MultiSynTech GmbH, Witten Germany). Wang resin preloaded with Fmoc-Glu(OtBu) was used as a solid support for the synthesis of **WT** and **L1**, rink amide MBHA resin was instead used for amidated peptides (**L2** and **L3**). Fmoc-amino acids (4-fold excess) were sequentially coupled to the growing peptide chain using DIPCDI/HOBt (N,N'-diisopropylcarbodiimide/1-hydroxybenzotriazole) (4-fold excess) as activating mixture for 1 h at room temperature. Cycles of deprotection of Fmoc (40% piperidine/N,N-dimethylformamide) and coupling with the subsequent amino acids were repeated until the desired peptide-bound resin was completed. N-terminal acetylation has been performed with acetic anhydride (0.5 M) with the presence of N-methylmorpholine (0.25 M) (3:1 v/v; 2 ml/0.2 g of resin) as the last synthetic step. The protected peptide-resin was treated with trifluoroacetic acid (TFA)/H_2O/triisopropylsilane 90:5:5; v/v; 10 mL per 0.2 g of resin[5] for 1.5 h at room temperature. After filtration of the resin, the solvent was concentrated *in vacuo* and the residue triturated with ethyl ether. Crude peptides were purified by preparative reversed-phase HPLC using a Water Delta Prep 3000 system with a Jupiter column C18 (250 × 30 mm, 300 A, 15 μm spherical particle size). The column was perfused at a flow rate of 20 ml/min with a mobile phase containing solvent A (water in 0.1% TFA), and a linear gradient from 0 to 80% of solvent B (60%, v/v, acetonitrile in 0.1% TFA) over 35 min for the elution of peptides. Analytical HPLC analyses were performed on a Beckman 116 liquid chromatograph equipped with a Beckman 166 diode array detector. Analytical purity of the peptides was assessed using a Zorbax C18 column (4.6 × 150 mm, 3 μm particle size) with the above solvent system (solvents A and B) programmed at a flow rate of 0.7 ml min^{-1} using a linear gradient from 0% to 100% B over 30 min. All analogues showed ≥ 95% purity when monitored at 220 nm. Molecular weight of final compounds was determined by a mass spectrometer ESI Micromass ZMD-2000.

12. References

1. D. Bellotti, M. Toniolo, D. Dudek, A. Mikołajczyk, R. Guerrini, A. Matera-Witkiewicz, M. Remelli and M. Rowińska-Żyrek, *Dalton Trans.*, 2019, **48**, 13740-13752.
2. L. D. Pettit and H. K. J. Powell, *The IUPAC Stability Constants Database*, Royal Society of Chemistry, London, 1992-2000.
3. H. Sigel and R. B. Martin, *Chem. Rev.*, 1982, **82**, 385-426.
4. N. L. Benoiton, *Chemistry of Peptide Synthesis*, Taylor & Francis, 2005.
5. N. A. Sole and G. Barany, *The Journal of Organic Chemistry*, 1992, **57**, 5399-5403.
